# Factors associated with catch‐up growth in early infancy in rural Pakistan: A longitudinal analysis of the women's work and nutrition study

**DOI:** 10.1111/mcn.12733

**Published:** 2018-11-13

**Authors:** Rebecca Pradeilles, Tom Norris, Elaine Ferguson, Haris Gazdar, Sidra Mazhar, Hussain Bux Mallah, Azmat Budhani, Rashid Mehmood, Saba Aslam, Alan D. Dangour, Elizabeth Allen

**Affiliations:** ^1^ Faculty of Epidemiology and Population Health London School of Hygiene & Tropical Medicine London UK; ^2^ School of Sport, Exercise and Health Sciences Loughborough University Loughborough UK; ^3^ Collective for Social Science Research Karachi Pakistan

**Keywords:** catch‐up growth, infant, length‐for‐age, Pakistan, predictors, rural

## Abstract

The adverse health impacts of early infant stunting can be partially ameliorated by early catch‐up growth. Few studies have examined predictors of and barriers to catch‐up growth to identify intervention points for improving linear growth during infancy. This study aimed to estimate the prevalence of, and factors associated with, catch‐up growth among infants in Pakistan. A longitudinal study of mother–infant dyads (*n* = 1,161) was conducted in rural Sindh province, with enrolment between December 2015 and February 2016 (infants aged 0.5–3 months), and follow‐up (*n* = 1035) between November 2016 and January 2017 (infants aged 9–15 months). The outcome was catch‐up growth (change in conditional length‐for‐age z‐scores >0.67 between baseline and endline). Associated factors were examined using multivariable logistic regression analyses. The prevalence of stunting was 45.3% at baseline and 60.7% at follow‐up. 22.8% of infants exhibited catch‐up growth over this period. Factors positively associated with catch‐up growth included maternal height (odds ratio (OR) = 1.08 [1.05–1.11]), household wealth (OR = 3.61 [1.90–6.84]), maternal (OR = 2.43 [1.30–4.56]) or paternal (OR = 1.46 [1.05–2.03]) education, and households with two or more adult females (OR = 1.91 [1.26–2.88]). Factors negatively associated with catch‐up growth were two (OR = 0.64 [0.45–0.89]) or three or more (OR = 0.44 [0.29–0.66]) preschool children in the household and the infant being currently breastfed (OR = 0.59 [0.41–0.88]). Catch‐up growth was exhibited among approximately a quarter of infants despite living in challenging environments associated with extremely high rates of early infant stunting. Several modifiable factors were identified that might represent suitable programme intervention points to off‐set early infant stunting in rural Pakistan.

**Key messages**

- Catch‐up growth was exhibited in 22.8% of infants over a 9‐month period.
- In those who exhibited catch‐up growth, length at endline was on average 4.08cm higher than in infants who did not show catch‐up growth, despite similar lengths at baseline.
- Factors positively associated with catch‐up growth included maternal height, household wealth, maternal and paternal education, and households with two or more adult females.
- Factors negatively associated with catch‐up growth were two or three or more preschool children in the household and the infant being currently breastfed.
- Factors identified represent intervention points to off-set early infant stunting in rural Pakistan.

## 1 INTRODUCTION

In low‐ and middle‐income countries (LMICs), growth impairment in utero is common and contributes to high rates of stunting in children under 5 years of age (Christian et al., 2013). Stunting in early life is associated with both short‐ and long‐term outcomes, including increased risks of morbidity and mortality, reduced cognitive development, and short adulthood stature. These latter outcomes also increase risks in later life of poor pregnancy outcomes and reduced adult work capacity (Crookston et al., 2013; Mason, Saldanha, & Martorell, 2012; Prendergast & Humphrey, 2014; Victora et al., 2008; Walker et al., 2011).

In geographical settings where stunting in early infancy is common, the physiological phenomenon of catch‐up growth can partially ameliorate these negative functional consequences, especially among infants who are born small for their gestational age (Darendeliler et al., 2008). Catch‐up growth (especially in small‐for‐gestational age infants) provides some protection against infections in early life (Victora, Barros, Horta, & Martorell, 2001); although its benefits extend beyond these short‐term outcomes. Differentiating between catch‐up in length and catch‐up in weight, however, is important because the two likely confer different risks for long‐term outcomes (developmental origins of health and disease paradigm). For example, catch‐up in weight, irrespective of size at birth, has a detrimental effect on later cardio‐metabolic outcomes (Baird et al., 2005; Ekelund et al., 2007; Ibanez, Ong, Dunger, & de Zegher, 2006; Monteiro & Victora, 2005; Soto et al., 2003). In contrast, rapid linear growth in the first two years of life, in LMICs, has been associated with increased adult height and number of years spent in school, without having negative health outcomes on rates of obesity or nutrition‐related noncommunicable diseases (Adair et al., 2013).

Given the favourable associations observed in LMICs between catch‐up growth in length and attained adult height, schooling outcomes, morbidity and mortality, understanding predictors of and barriers to catch‐up growth is important to inform intervention design. However, in settings where stunting in early infancy is common, there is limited evidence on the extent to which catch‐up growth in length occurs in the first year of life and on the factors associated with it. Therefore, the objectives of the current analyses were to estimate the percentage of infants who experienced catch‐up growth in length (hereinafter termed “catch‐up growth”) in a rural population in Pakistan, and identify factors associated with it. In particular, we examined the association of sociodemographic‐, nutrition‐, hygiene‐, agricultural‐, and health‐related factors with the presence of catch‐up growth in the first year of life.

## 2 METHODS

### 2.1 Study design

As part of the Leveraging Agriculture for Nutrition in South Asia (LANSA) programme, a longitudinal study (Women's Work and Nutrition) was conducted among mother–infant dyads in perennial canal irrigated rural areas of Sindh province, Pakistan, from December 2015 to February 2016 (baseline) and from November 2016 to January 2017 (endline). The primary aim of the overall study was to investigate the implications of agricultural labour by women on the nutritional status and health of women and their offspring. The sample size (*n* = 1,000 dyads) was chosen to detect a difference in maternal body mass index of 0.18 for every additional hour worked with 80% power at a 5% level of significance. This particular study is a secondary analysis of the available data to explore predictors of catch‐up growth in infants, in their first year of life.

Ethical approval, for the study, was obtained from the London School of Hygiene and Tropical Medicine (certificate number 9647) and from the Internal Ethics Review Committee of the Collective for Social Science Research in Pakistan. The respondent (i.e., primary caregiver) gave verbal informed consent for herself and her child to participate in the study. The study was conducted according to guidelines laid down in the Declaration of Helsinki.

### 2.2 Sampling

Participants were selected via systematic random cluster sampling. In the first phase, all administrative villages in Sindh province's districts and sub‐districts with perennial canal irrigation were selected (2,911 of 5,775 administrative villages). The reported populations of these villages ranged from <100 to >34,000 and we therefore excluded villages if their population, as reported in the 1998 census, was below the 10th and above the 90th percentiles of estimated village sizes (i.e., populations <1,000 or > 7,800). A random sample of villages was used to recruit at least 1,000 mother–infant dyads. All eligible mother–infant dyads living in the selected villages were invited to participate in the study. The inclusion criteria were: infant aged ≥2 weeks and ≤ 12 weeks of age on the day of the first interview, healthy infant without congenital malformations that would impact on their ability to eat and grow, a primary caregiver (i.e., the biological mother) who stated that she planned to reside in the study area over the next 10 months and a singleton birth. Detailed information on the recruitment process of mother‐infant dyads is provided in **supplementary appendix 1**.

### 2.3 Questionnaire, spot observations, and anthropometric measurements

An interviewer‐administered questionnaire was carried out at both baseline and endline using electronic data capture (Samsung tab‐4) to obtain information relating to: socio‐demographic characteristics, household food insecurity, 24h food consumption, maternal agricultural work history (during and after pregnancy), maternal and child health, nutrition knowledge, and water/sanitation practices. At both baseline and endline, two measurements of maternal and infant weight and height/length were collected by trained fieldworkers and according to standard procedures (WHO, 2006). For weight and height/length, a third measurement was taken if the difference between the first two measurements was above a predefined threshold (i.e., >0.7 cm for maternal height, >0.5 kg for maternal weight, > 0.7 cm cm for infant length, and > 0.1 kg for infant weight); and an average of the two closest measurements was used. Details regarding the methods of obtaining anthropometric measurements are included in **supplementary appendix 2**. Information on birth weight was collected but not used as the majority of births in rural Pakistan take place in a noninstitutional setting where children are less likely to be weighed. In this sample, only 7.0% of infants had been weighed at birth.

### 2.4 Data analyses

#### 2.4.1 Anthropometric indices/variables

Sex‐specific length‐for‐age z‐scores (LAZ) were generated relative to the WHO growth standards (WHO, 2006). A z‐score of <−6 SD or >+6 SD were considered as biologically implausible, and cases with such values were removed. Infants were classified as stunted and severely stunted if their LAZ were <−2 SD and <−3 SD, respectively (WHO, 2006).

To avoid problems with regression to the mean (RTM), conditional growth measures were calculated by performing regressions of z‐score_(time2)_ on z‐score_(time1)_, such that:

$$\text{Conditional growth}=z-{\text{score}}_{\left(\text{time2}\right)}–\left(c+\beta^{*}z-{\text{score}}_{\left(\text{time1}\right)}\right)$$

where c and β are the intercept and slope coefficient, respectively, of regressing z‐score_(time2)_ on z‐score_(time1)_. The residuals from this regression represent the portion of size at time2, which is uncorrelated with size at time1 and thus represents conditional growth during that period, independent of RTM (Cameron, Preece, & Cole, 2005; Johnson, 2015). Based upon the widely used definition of catch‐up growth proposed by Ong et al. (2000), instances where z‐score residuals were greater than +0.67 (the width of a centile band on a standard growth chart) were taken to indicate “catch‐up growth.” The classification of catch‐up growth as a dichotomous variable represents a clinically interpretable outcome (i.e., the space between two centiles on a standard growth chart) and has been used widely in research, showing associations with later outcomes (Ong, Ahmed, Emmett, Preece, & Dunger, 2000; Ong & Loos, 2006; Raaijmakers et al., 2017; Taal, Steegers, Hofman, & Jaddoe, 2013).

Associations between the presence or absence of catch‐up growth and a range of exposures (e.g., individual‐ and household‐level factors, nutrition‐related factors, agriculture‐related factors, and health‐related factors) were investigated. The exposure variables included in the analysis are described below. Exposure variables were predominantly collected at baseline; however, these were supplemented by a number of relevant endline variables which are related to exposures experienced during the intermittent period between baseline‐endline (e.g., infant feeding practices and household food insecurity). Further information on the definitions and generations of these indicators can be found in **supplementary appendix 3**.

#### 2.4.2 Individual‐ and household‐level factors

Individual level variables included in the analysis were maternal age, maternal height, and infant sex. Household‐level variables included maternal/paternal education; household wealth index; household food insecurity access scale (Coates, Swindale, & Bilinsky, 2007); water, sanitation, and hygiene index (WASH); maternal occupation; number of children under five (including the reference child); and number of adult females per household (≥14 years).

#### 2.4.3 Nutrition‐related factors

The infant and young child minimum dietary diversity score was generated for infants (WHO, 2008), using reported intakes of foods and beverages during the past 24‐hours. Other nutrition‐related factors explored in the analysis were maternal nutrition knowledge, current breastfeeding status, and whether the mother initiated breastfeeding early.

#### 2.4.4 Agricultural‐related factors

Cotton harvesting performed in the past season (post‐pregnancy). This variable was included as we hypothesized that maternal engagement in labour‐intensive agricultural activities, such as cotton harvesting, will compromise catch‐up growth because mothers will have less time for childcare and adequate feeding practices.

#### 2.4.5 Health‐related factors

Information on both maternal health (e.g., depression) and infant health (e.g., diarrhoea, cough, and fever experienced in the past 15 days and vaccination status) was explored in the analysis.

### 2.5 Statistical analyses

Descriptive statistics were generated for individual, household, nutrition, agricultural, and health‐related factors.

Hypothesized models of pathways related to catch‐up growth were drawn based on the available literature and discussion with experts. Consensus from these meetings was captured in the form of directed acyclic graphs (DAGs) (supplementary appendix 4), which are causal diagrams that provide a method for visualising relationships between variables in order to build unbiased (or less biased) causal models (Greenland, Pearl, & Robins, 1999; Moodie & Stephens, 2010). DAGs provide researchers with a set of variables for which adjustment is necessary or unnecessary (e.g., for factors on the causal pathway between the exposure and outcome, i.e. mediators).

Associations between individual, household, nutritional, health, and agricultural‐related factors (i.e., the main exposure variables) and catch‐up growth were examined using univariable and multivariable logistic regression. A DAG was produced for each separate exposure in relation to catch‐up growth resulting in the construction of a series of separate multivariable logistic regression models. The variables included in the multivariable models were retained based on the information provided by the DAGs (**supplementary appendix 4**). Some exposures not identified as confounding factors were included in all multivariable models a priori due to their known effect on infant growth (i.e., maternal age, maternal height and child's sex (Addo et al., 2013; Fall et al., 2015; Lejarraga, 2002). Variables on the causal pathways (i.e. mediators) were not adjusted for. Robust standard errors were used to account for clustering at the village level.

In order to assess the validity of results from a complete case analysis, baseline measures of children with and without endline LAZ data were compared (**supplementary appendix 5**). Sensitivity analyses were also carried out to explore the possible impact of missing catch‐up growth data on observed relationships (**supplementary appendix 6**). This was done by rerunning the analyses replacing all missing catch‐up growth data with catch‐up growth = 1 and then again replacing all missing catch‐up growth data with catch‐up growth = 0.

All analyses were conducted using Stata/IC (version 14.1). The Type I error risk was set at 0.05.

## 3 RESULTS

The sample of mother–infant dyads at endline was 1,035, which represented 89.1% of the initial sample (*n* = 1161). The reasons for drop outs (*n* = 126) were: infant death (*n* = 26; 20.7%), migration (*n* = 45; 35.7%), non‐availability during team visit (*n* = 38; 30.1%), refusals (*n* = 9; 7.1%), enumerator error (n = 4; 3.2%), and unidentified reasons (n = 4; 3.2%). The sample of infants with complete data for LAZ at both baseline and endline was 1,024, which was further reduced to 1,008 after removal of outliers (i.e., *n* = 9 at baseline and *n* = 11 at endline; **supplementary appendix 7**).

Compared with those who did not have LAZ data at endline, there was weak evidence that in those infants with data, the prevalence of stunting at baseline was lower (44% vs. 54%; *p* = 0.04), and the proportion with an educated mother was higher (20% vs. 13%; p = 0.04). There were no other differences in infant, maternal, and household characteristics of infants who did and did not have complete data (**supplementary appendix 5**).

85.7% of infants were currently breastfed (when aged 9–15 months) with 29.4% of infants having received an early initiation of breastfeeding **(**Table 1 **)**. 9.3% of infants achieved minimum dietary diversity (i.e., consumption of at least four groups out of seven) when aged 9–15 months. Parents' level of education was low (80.0% of mothers in the sample had no formal education vs. 47.1% of fathers). More than half (52.3%) of women were engaged in the agricultural sector, with 36.2% involved in cotton‐harvesting in the past season. The household food insecurity level was high with 70.7% of households classed as food insecure (44.6% of households were classed as severely food insecure). Maternal nutrition knowledge and WASH practices were low, with 71.0% and 78.2% of mothers scoring less than six (both out of 11) on the nutrition knowledge and WASH scores, respectively. 73.3% of households had two infants or more, whereas 68.5% of households had two or more adult females.

**Table 1:** Socio‐demographic characteristics for those with complete outcome data at both baseline and endline (*n* = 1,008)

	Complete infant baseline and endline data (*n* = 1,008)	
	Total *n*	
Infant characteristics		
Sex	1,008	
Female (%)	505	50.1
Age at baseline (months)	1,008	1.6 (0.7)
Age at endline (months)	1,008	12.2 (0.7)
Current breastfeeding status (endline)	1,002	
Yes (%)	859	85.7
Early initiation of breastfeeding	1,003	
Yes (%)	295	29.4
Child minimum dietary diversity	1002	
Consuming at least 4 food groups (%)	93	9.3
Maternal, paternal, and household characteristics		
Maternal age (years)	970	27.0 (23.0, 32.0)
Maternal height (cm)	1,008	152.6 (5.4)
Maternal education (%)	1,008	
No formal education	806	80.0
Primary school education	126	12.4
>primary school education	77	7.6
Paternal education (%)	999	
No formal education	470	47.1
Primary school education	189	18.9
>primary school education	340	34.0
Maternal occupation (%)	995	
Unemployed	331	33.3
Agriculture‐related employment	520	52.3
Nonagriculture related employment	144	14.5
Cotton‐harvesting in the past season (%)	1,002	
None	639	63.8
1–2 months	176	17.6
≥2 months	187	18.6
Maternal, paternal, and household characteristics		
Nutrition knowledge score (out of 11; %)	1,002	
1 (0–3 points)	403	40.2
2 (4–5 points)	309	30.8
3 (≥6 points)	290	28.9
WASH index (out of 11; %)	1,006	
1 (0–3 points)	486	48.3
2 (4–5 points)	301	29.9
3 (≥6 points;i.e., good practices)	219	21.8
Household food insecurity (%)	994	
Food secure	291	29.3
Mildly food insecure	67	6.7
Moderately food insecure	193	19.4
Severely food insecure	443	44.6
Number of adult females (≥14 years; %)	1,008	
1	318	31.5
2	310	30.8
3 or more	380	37.7
Number of preschool children (%)	1,008	
1	269	26.7
2	402	39.9
3 or more	337	33.4

*Note*. WASH: water, sanitation, and hygiene. Continuous variables are summarized as mean (*SD*) or median (IQR). Categorical variables are summarised using n (%)

At baseline, 45.3% of infants (aged 0.5–3 months) were stunted, which increased to 60.7% at endline (aged 9–15 months). The prevalence of severe stunting (LAZ < −3) at endline was 27.4% (data not shown). Despite having similar rates of stunting at baseline, infants who exhibited catch‐up growth had a significantly lower prevalence of stunting at endline compared with those who did not experience catch‐up growth (14.3% vs. 74.2%, *p* < 0.001) **(**Table 2 **)**. In the 22.8% of infants who exhibited catch‐up growth, length‐for‐age z‐scores at endline were on average 1.63z (1.48; 1.77) higher than in those who had shown no catch‐up growth (despite similar lengths at baseline): equivalent to a difference of 4.08 cm (3.68; 4.48) in length.

**Table 2:** Infant anthropometric characteristics by catch‐up growth status (*n* = 1,008)

	Catch‐up growth (*n* = 230)	Noncatch‐up growth (*n* = 778)	Difference^a^ (catch‐up–noncatch‐up growth)
Length at baseline (cm; mean; SD)	52.66 (3.99)	52.71 (3.44)	−0.05 (−0.57; 0.48)
Length‐for‐age z score baseline (mean; SD)	−1.84 (1.53)	−1.78 (1.31)	−0.06 (−0.26; 0.14)
Stunted at baseline (%(n))	45.22 (104)	43.96 (342)	1.26 (−6.06; 8.57)
Length at endline (cm; mean; SD)	72.48 (2.46)	68.40 (2.81)	4.08 (3.68; 4.48)
Length‐for‐age z score endline (mean; SD)	−1.06 (0.88)	−2.69 (1.02)	1.63 (1.48;1.77)
Stunted at endline (%(n))	14.35 (33)	74.16 (577)	−59.82 (−65.29; −54.34)

*Note*. *SD*: standard deviation.

a Categorical variables: chi‐squared test; continuous variables: student's t‐tests for mean differences (95% CI) and quantile regression (50th centile) for differences in medians.

In the univariable analysis, factors positively associated with catch‐up growth were: maternal height, household wealth, maternal or paternal education, a household adopting positive WASH practices, a diverse infant diet (consumed ≥4 food groups; minimum diet diversity), and the presence of two or more adult females in the household (vs. one) (Table 3). Factors negatively associated with catch‐up growth were: a household having two or more preschool children (vs. one), whether the mother was employed in agricultural‐related work (vs. not working), whether the household was moderately or severely food insecure, whether the mother was involved in cotton‐harvesting 2 months or more in the past season (vs. not involved), and whether the child was currently being breastfed. Health‐related factors (i.e., maternal depression, child's health, and vaccination status) did not show any association with catch‐up growth (data not shown).

**Table 3:** Factors associated with catch‐up growth (defined as change in length‐for‐age z‐score > 0.67) from the univariable and multivariable logistic regression analyses

	Crude		Adjusted	
	OR	CI (95%)	OR	CI (95%)
	*p* = 0.805			
Maternal age (years)	0.99	0.97; 1.02		
	*p* < 0.0001			
Maternal height (cm)	1.08	1.05; 1.11		
Infant sex	*p* = 0.226			
Male	Ref	_		
Female	0.82	0.60; 1.13		
Maternal education	*p* < 0.0001		*p* < 0.0001^a^	
Not educated (ref)	Ref	_	Ref	_
Primary school	2.46	1.62; 3.76	2.46	1.62; 3.76
Middle, secondary, and higher education	2.61	1.39; 4.89	2.43	1.30; 4.56
Paternal education	*p* = 0.012		*p* = 0.046^b^	
Not educated (ref)	Ref	_	Ref	_
Primary school	1.05	0.68; 1.62	1.07	0.68; 1.69
Middle, secondary, and higher education	1.53	1.11; 2.09	1.46	1.05; 2.03
Household wealth index	*p* < 0.0001		*p* = 0.0017^c^	
Poorest (ref)	Ref	_	Ref	_
Poor	1.56	0.90; 2.72	1.77	1.01; 3.11
Middle	2.06	1.10; 3.86	1.91	0.96; 3.82
Wealthy	2.22	1.27; 3.90	2.16	1.17; 3.99
Wealthiest	4.03	2.35; 6.93	3.61	1.90; 6.84
Maternal occupation	*p* = 0.007		*p* = 0.55^d^	
Not working (ref)	Ref	_	Ref	
Non‐agricultural related work	0.99	0.66; 1.50	0.86	0.61; 1.20
Agricultural related work	0.63	0.46; 0.86	1.08	0.66; 1.77
Household FI (4 categories)	*p* < 0.0001		*p* = 0.41^e^	
Food secure (ref)	Ref	_	Ref	
Mildly food insecure	0.71	0.40; 1.26	0.66	0.35; 1.24
Moderately food insecure	0.65	0.44; 0.96	0.83	0.52; 1.32
Severely food insecure	0.49	0.34; 0.71	0.74	0.48; 1.12
Number of adult females (≥14 years)	*p* = 0.001		*p* = 0.01^f^	
1 (ref)	Ref	_	Ref	_
2	2.08	1.42; 3.04	1.91	1.26; 2.88
≥3	1.60	1.10; 2.32	1.28	0.86; 1.90
Number of children under 5 years	*p* = 0.0001		*p* = 0.0003^g^	
1 (ref)	Ref	_	Ref	_
2	0.62	0.44; 0.87	0.64	0.45; 0.89
≥3	0.43	0.29; 0.63	0.44	0.29; 0.66
Household level factors				
WASH index (3 categories; 12 variable score)	*p* = 0.001		*p* = 0.21^h^	
1 (0–3 points; ref)	Ref	_	Ref	
2 (4–5 points)	1.48	1.08; 2.03	1.23	0.85; 1.77
3 (≥6 or more;i.e., good practices)	2.04	1.37; 3.04	1.39	0.95; 2.05
Nutrition‐related factors				
Child minimum dietary diversity (binary)	*p* = 0.010		*p* = 0.28^i^	
No (ref)	Ref	_	Ref	‐
Yes	1.81	1.15; 2.83	1.41	0.76; 2.61
Breastfeeding status	*p* = 0.002		*p* = 0.009^j^	
No (ref)	Ref	_	Ref	_
Yes	0.59	0.42; 0.82	0.59	0.41; 0.88
Early initiation of breastfeeding	*p* = 0.93		*p* = 0.40^k^	
No (ref)	Ref	_	Ref	_
Yes	0.98	0.74; 1.32	1.14	0.84; 1.53
Nutrition knowledge score (out of 11)	*p* = 0.14		*p* = 0.52^l^	
1 (0–3 points; ref)	Ref	_	Ref	
2 (4–5 points)	1.29	0.95; 1.76	1.19	0.85; 1.69
3 (≥6 points)	1.51	1.00; 2.27	1.06	0.67; 1.68
Agriculture‐related factors				
Cotton‐harvesting past season (3 categories)	*p* = 0.03		*p* = 0.03^m^	
No cotton harvesting (ref)	Ref	_	Ref	_
1–2 months	1.05	0.67; 1.63	1.42	0.92; 2.17
≥2 months	0.60	0.39; 0.90	0.74	0.48; 1.13

*Note*. CI: confidence interval.

WASH: water, sanitation, and hygiene.

a adjusted for infant sex, maternal height and maternal age (base model) (*n*=964).

b adjusted for variables in the base model (*n*=955).

c adjusted for paternal education, maternal education and variables in the base model (*n*=927).

d adjusted for paternal education, maternal education, SES and variables in the base model (*n*=914).

e adjusted for cotton picking, SES, maternal/paternal education, number of children <5, number adult females, base model (*n*=919).

f adjusted for paternal/maternal education, SES, base model (*n*=927).

g adjusted for paternal/maternal education, SES, base model (*n*=927).

h adjusted for cotton picking, SES, maternal/paternal education, number of adult females, nutrition knowledge (*n*=927).

i adjusted for cotton picking, HFIAS, SES, maternal depression, maternal/paternal education, number adult females, number of children under 5, nutrition knowledge score, base model (*n*=927).

j adjusted for SES, maternal education, paternal education, nutrition knowledge and variables in the base model (*n*=927).

k adjusted for SES, maternal education, paternal education, nutrition knowledge and variables in the base model (*n*=923).

l adjusted for maternal education, SES and variables in the base model (*n*=936).

m adjusted for paternal education, maternal education, SES and variables in the base model (*n*=927).

Supplementary appendix 4 provides rationale for the model building process described above.

The multivariable analysis (Table 3) showed that after adjusting for a set of putative confounders identified in the DAGs, maternal height (OR = 1.08 [1.05; 1.11]) and maternal and paternal education (OR_mother_ = 2.43 [1.30; 4.56] and OR_father_ = 1.46 [1.05; 2.03] for “middle, secondary, or higher education” vs. none) remained positively associated with catch‐up growth. Furthermore, the household wealth index (OR = 2.16 [1.17; 3.99] and OR = 3.61 [1.90; 6.84] for the “wealthy” and “wealthiest” households, respectively, compared with the poorest) and the number of adult females (OR = 1.91 [1.26; 2.88] for households with two adult females vs. one) also maintained their positive associations upon adjustment. Number of preschool children in the household (OR = 0.64 [0.45; 0.89] for two children vs. one and OR = 0.44 [0.29; 0.66] for three or more children vs. one), and breastfeeding status (OR = 0.59 [0.41; 0.88]) remained negatively associated with catch‐up growth. Maternal occupation, cotton‐harvesting performed in the past season, household food insecurity, WASH practices, and the child minimum dietary diversity were no longer associated.

Replacing missing catch‐up growth measures with 1 and then with 0 did not alter the conclusions from the complete case analysis (**supplementary appendix 6**).

## 4 DISCUSSION

The high prevalence of stunting among young infants in rural Pakistan increased from 45.3%, when the infants were 0.5 to 3 months of age to 60.7%, when they were 9 to 15 months of age. However, even in this environment associated with high rates of stunting, close to one quarter of infants experienced catch‐up growth. At endline, the difference in LAZ between infants who had and had not exhibited catch‐up growth was 1.63 z‐scores, which translates to a difference of 4.08 cm in length. Further, the prevalence of stunting at 9–15 months of age was approximately five times lower in those who had demonstrated catch‐up growth compared with those who had not (14.4% vs. 74.2%), despite similar mean length‐for‐age z‐scores at baseline.

To our knowledge, this is the first study to investigate household, nutrition, agriculture, and health‐related factors associated with catch‐up growth in a representative sample of infants in South Asia. The factors associated with catch‐up growth were mainly socio‐demographic. Maternal education, paternal education, the household wealth index, and the number of adult females per household were all positively associated with infant catch‐up growth, whereas having a sibling of preschool age and breastfeeding were negatively associated with it. Maternal height was also positively associated with catch‐up growth, indicating the role of a genetic potential for growth. Unexpectedly, catch‐up growth was not associated with health‐related factors, WASH practices or labour‐intensive agricultural work (i.e. cotton harvesting). Perhaps, our short‐term indicators of infant health status (i.e., illness in the previous 14 days) did not adequately capture long‐term health status; and, in rural LMIC environments, community‐wide changes in sanitation might be required to improve infant growth (Headey & Hoddinott, 2015). Labour‐intensive agricultural work, during pregnancy, was negatively associated with LAZ in these infants at baseline (Pradeilles et al., 2018 [unpublished]). Perhaps after birth, family support, when agricultural workload demands are high, buffers the negative consequences of an absent mother. The positive association between catch‐up growth and the number of female adults in the household provides evidence of the important role of family support.

These results are consistent with other studies (Bavdekar, Vaidya, Bhave, & Pandit, 1994; Nguyen et al., 2017). In India, catch‐up growth, in low birth weight survivors, was positively associated with parental height, weight, and socio‐economic status (Bavdekar et al., 1994). In Bangladesh and Vietnam, intervention‐related improvements in socio‐economic status, maternal education, and birth weight were negatively associated with the prevalence of stunting in 24–48 month old children (Nguyen et al., 2017). However, in contrast to our study, intervention‐related improvements in food security, hygiene, and maternal nutrition knowledge (in Vietnam only) were associated with change in LAZ. Inter‐study differences in study design and timeframe likely account for these inconsistencies. In Bangladesh and Vietnam, participants were enrolled in an intervention programme and catch‐up growth was not measured (Nguyen et al., 2017).

The reason catch‐up growth was negatively associated with breastfeeding (at endline), in our study, is not clear. There is mixed evidence regarding the effect of breastfeeding (vs. formula feeding) on infant growth. Although data from observational studies conducted in well‐off environments suggest that formula‐fed infants grow faster than healthy breastfed infants (Dewey, 1998; Dewey et al., 1995), this has not been observed in randomised controlled trials (Kramer et al., 2002). In rural disadvantaged environments, bacterial contamination of milk or the practice of diluting formula milk would likely impair growth. In our study, the percentage of formula‐fed infants in the group experiencing catch‐up growth was low (3.9%). Infant age, sex, and household socio‐economic status, which were controlled in the analyses, also do not account for this observed finding. A plausible contributing factor towards this finding may be that infants who are currently breastfed (either exclusively or not), receive poorer complementary feeding both in terms of quality and quantity compared with those who are not breastfed. Indeed, we observed that only 7.7% and 5.8% of infants who were breastfed achieved minimum dietary diversity and the minimum meal frequency, respectively, vs. 17.6% and 17.0% in nonbreastfed infants. As the prevalence of breastfeeding was higher among those who did not catch‐up, this may contribute to the inverse finding we observed between breastfeeding status and catch‐up growth. We also noticed that of those who were breastfed, 12.1% (mean age = 12.2 months) had a value of 0 on the dietary diversity score and minimum meal frequency, suggesting that these infants were exclusively breastfed the day of the recall. Given the age at which this was collected, this represents a relatively high proportion of exclusive breastfeeding, again suggesting that this sample of infants may not have been in receipt of adequate complementary feeding. Although the proportion of exclusive breastfeeding was higher in those who did not catch‐up, the difference (with those who did) was not significant.

To our knowledge, this is the first study to use a well‐accepted definition of catch‐up growth to identify predictors that are associated with a clinically relevant change in LAZ. Other studies have focused on change in length/height and weight‐for‐age as continuous measures (Adair et al., 2013; Khadilkar et al., 2016; Pelletier, Frongillo Jr, & Habicht, 1993) or have not used a definition of catch‐up growth that accounts for correlation between subsequent z‐scores (Bavdekar et al., 1994). We have defined catch‐up growth as a change in conditional z‐scores >0.67, thus accounting for RTM. Although we believe this approach is more appropriate than the original definition of catch‐up growth as a change in unconditional z‐scores of >0.67, that is, z‐score_2_–z‐score_1_ (Ong et al., 2000), it is likely that this would result in fewer infants being classified as exhibiting catch‐up growth (Eckhardt, Gordon‐Larsen, & Adair, 2005). However, as our definition is based on the residuals obtained after the regression of z‐score_2_ on z‐score_1_, if the residuals are normally distributed (as is the assumption of linear regression), we would expect 25% of the sample to have residuals >0.67z and thus demonstrate catch‐up growth. There is no guarantee that residuals would be normally distributed however and the prevalence observed in this sample (22.8%) represents the slight positive skewness of the residual distribution.

Other strengths of our study are the sampling frame, large sample size (*n* = 1008), and the use of DAGs to guide statistical model structure. We can extrapolate the findings to similar canal‐irrigated rural areas of Pakistan. The sensitivity analyses gave similar results to the complete case analyses, further supporting the robustness of the findings. The large sample size and comprehensive dataset also meant relationships between selected exposures and catch‐up growth were investigated while considering the underlying causal network (e.g., socio‐economic status water, sanitation, and hygiene practices and other maternal, paternal, and infant‐related factors). The use of DAGs, which are based on an understanding of the causal network linking the variables in the analysis, provides a less biased estimate of the effect of changing levels of exposures on catch‐up growth via appropriate adjustment of confounding factors. We are, however, cautious not to interpret any effect as “causal” as we cannot exclude the possibility of the presence of both residual confounding and measurement error.

The absence of birthweight data is the main weakness of this study. We could not investigate catch‐up growth relative to size at birth because our baseline measurements were made when the infants were between 2 and 12 weeks of age. Additionally, some of the exposure variables were only collected at endline, which coincided with the collection of LAZ and thus temporality could not be ensured. Finally, our key indicator of labour‐intensive agriculture work (cotton‐harvesting) was subject to recall bias. Women were asked to provide information on the number of months worked and then specify the number of days and hours worked during the cotton harvesting season, which ran from July to November. Measurement error might have attenuated the relationship between catch‐up growth and cotton‐harvesting.

Nevertheless, given the nutritional landscape of Pakistan (i.e., high rates of stunting and morbi‐mortality), the current study fills an important gap with regards to factors that could improve linear growth in the first year of life, which is a critical period for later health outcomes (e.g., subsequent linear growth and cardio‐metabolic outcomes) and the development of human capacity (Adair et al., 2013). Together, our results and others (Bavdekar et al., 1994; Headey, Hoddinott, Ali, Tesfaye, & Dereje, 2015; Headey & Hoddinott, 2015; Nguyen et al., 2017) highlight the importance of national level improvements in wealth status and parental education for improving child growth and identify potential target points that can be leveraged to improve infant linear growth in this particular setting.

In conclusion, this study generates new evidence with regards to catch‐up growth patterns and its associated factors, among infants living in rural Pakistan. Our analysis showed that a substantial proportion of infants experienced catch‐up growth despite living in a challenging environment associated with extremely high rates of stunting. It is likely, given the evidence which shows that in LMICs, catch‐up growth in length improves adult stature, schooling and hence human capital (Adair et al., 2013), that in rural Pakistan, where levels of stunting, wasting, morbidity, and mortality are high, catch‐up growth will likely prove beneficial to long term health.

The modifiable environmental factors associated with catch‐up growth in this setting, where women face an important burden of work and care, emphasise the need to develop and/or strengthen existing nutrition‐sensitive programmes (e.g., programmes targeting maternal and paternal education, socio‐economic status, and agriculture income generation), alongside sensible policy responses (e.g., ensuring availability and access to high quality child care for working mothers). Such interventions might help support linear catch‐up growth in infancy (a critical window of opportunity within the first 1,000 days paradigm), potentially improving subsequent health outcomes. Tackling wider and proximal determinants of linear catch‐up growth will help achieve the internationally‐agreed targets for reducing childhood stunting (<5 years of age) by 2030, as part of the Sustainable Development Goals.

## CONFLICTS OF INTEREST

The authors declare that they have no conflicts of interest.

## CONTRIBUTIONS

HG and EF designed the research with the help of EA, ADD, HBM, and RM. SM, AB, HBM, RM, and SA conducted the research. SM and RP managed the data. RP and TN performed statistical analysis with the help of EA and RP, and TN wrote the first draft of the paper. All authors took responsibility for final editing, reviewing, and approval of the manuscript.

## Supporting information


**Data S1.** Sampling
**Supplementary Appendix 2.** Information on anthropometric measurements
**Supplementary Appendix 3.** Variables and indices
**Supplementary Appendix 4.** Hypothesized models of pathways related to catch‐up growth represented on a directed acyclic graph (DAG)
**Supplementary Appendix 5.** Sample characteristics for infants with and without endline outcome data
**Supplementary Appendix 6.** Sensitivity analyses
**Supplementary Appendix 7.** Sample flow chart
