# Supplementary material for: Factors associated with catch‐up growth in early infancy in rural Pakistan: A longitudinal analysis of the women's work and nutrition study
Source: Matern Child Nutr. 2018 Nov 13;15(2):e12733. doi: 10.1111/mcn.12733 (PMC6587826; doi:10.1111/mcn.12733)
Supplement: Supplementary file 1 — Data S1. Sampling Supplementary Appendix 2. Information on anthropometric measurements Supplementary Appendix 3. Variables and indices Supplementary Appendix 4. Hypothesized models of pathways related to catch‐up growth represented on a directed acyclic graph (DAG) Supplementary Appendix 5. Sample characteristics for infants with and without endline outcome data Supplementary Appendix 6. Sensitivity analyses Supplementary Appendix 7. Sample flow chart [file MCN-15-e12733-s001.docx]

**Supplementary appendix**

**Appendix 1: Sampling**

A sample size of 1000 dyads was chosen to detect a difference in maternal BMI of 0.18 for every additional hour worked with 80% power at a 5% level of significance (National Institute of Population Studies, 2013). This sample size also provides adequate power to explore factors associated with maternal and infant nutritional status.

Participants were selected via systematic random cluster sampling. In the first phase, all administrative villages with perennial canal irrigation were selected (2,911 of 5,775 administrative villages). Villages with perennial canal irrigation were chosen as the study site because women in these villages are frequently involved in commercial agriculture, including cotton harvesting. The reported populations of these villages ranged from <100 to >34,000 and we therefore excluded villages if their population, as reported in the 1998 census, was below the 10^th^ and above the 90^th^ percentiles of estimated village sizes (i.e., populations < 1000 or > 7800). All eligible villages were listed in alphabetical ascending order by district (n=2329 villages); and every 37^th^ village was selected from a randomly selected start point in the list to provide an estimated sample size (based on birth rates) of 1000 mother-infant dyads. All dyads living in the selected villages were invited to participate in the study if they met the following inclusion criteria: (i) infant ≥2 weeks and ≤ 12 weeks of age on the day of the first interview; (ii) healthy infant without congenital deformations that would impact on their ability to eat; (iii) the primary caregiver (i.e. the biological mother) intends to reside in the study area over the next 10 months and (iv) a singleton birth.

To recruit these dyads, a community profiling procedure was used, in which local key informants, including: health workers, midwives, doctors, paramedics, and local authorities identified recent births. In the first stage, key informants were asked to list exhaustively all kinship groups/castes and localities within the village and then to list all births within the past three months from within those castes/localities. Probes were used to minimise omissions. A fieldworker visited each listed mother to confirm eligibility and probe for other births within the locality.

**References**

National Institute of Population Studies (NIPS) [Pakistan] and ICF International. Pakistan Demographic and Health Survey 2012–13. Islamabad, Pakistan and Calverton, Maryland, USA: NIPS and ICF International 2013.

**Appendix 2: Information on anthropometric measurements**

At both baseline and endline, two serial measurements of maternal and infant weight and height/length were collected following standard procedures (WHO, 2006) by trained fieldworkers who were selected on the basis of their technical error of measurement results at the end of a 5-day training. After removing shoes and heavy clothing, maternal weight was measured to the nearest 0.1kg using digital electronic scales (at baseline, a Tanita digital bathroom scale was used whilst at endline the following model was used CAMRY EB9-4A Series); maternal height was measured to the nearest 0.1cm using a portable stadiometer (Seca 213). At baseline, infant weight was measured to the nearest 0.01kg using digital electronic scales (LAICA weight scale for babies) and infant length was measured using an infantometer (Seca 416) to the nearest 0.1cm. At endline, the child was weighed in the mother’s arms and measured to the nearest 0.1kg using digital electronic scales (CAMRY EB9-4A Series). A third measurement was taken if the difference between the first two measurements was above a pre-defined threshold (i.e. >0.7cm for maternal height; >0.5kg for maternal weight; > 0.7cm cm for infant length; > 0.1kg for infant weight); and an average of the two closest measurements was used. No adjustments were made to infant weight at baseline as babies were naked/wearing very light clothing when age 2-12 weeks. At endline, adjustments were made according to the type of clothing recorded during data collection.

**References**

WHO Multicentre Growth Reference Study Group. WHO Child Growth Standards: length/height-for-age, weight-for-age, weight-for-length, weight-for-height and body mass index-for-age. Methods and development. Geneva: World Health Organization 2006.

**Appendix 3: Variables and indices**

| **Individual and household-level variables** | |
| --- | --- |
| **Household wealth index** (baseline) | A household wealth index was created using factor analysis applied to proxy indicators of the household environment (ownership of consumer durables; house ownership; land ownership; main source of energy for cooking; livestock ownership; electricity; source of drinking water and type of toilet facilities; number of rooms used for sleeping; type of materials used for floor, roof and walls). The wealth index was categorized into quintiles, with the 1^st^ quintile representing the relatively poorest. Internal validity was assessed by tabulating ownership of durable assets and housing characteristics by SES quintile. |
| **Maternal & paternal education** (baseline) | Neither maternal nor paternal education were included in the creation of the household wealth index because of their known independent effect on nutrition and health outcomes. Three categories were created for education: not educated; primary school; middle, secondary and higher education. |
| **Household food insecurity access scale (HFIAS)** (endline) | Based on answers to questions on anxiety and uncertainty about the household food supply and insufficient food quality experienced in the past 30 days, a Household Food Insecurity Access Scale (HFIAS) score was created (continuous score ranging from 0 to 27) (Coates & Bilinsky 2007). A categorical variable was also created to represent different levels of food security (food secure; mildly food secure; moderately food secure; severely food secure). |
| **Water, sanitation and hygiene index (WASH)** (baseline) | A summative index ranging from 0-12 was created to represent water, sanitation and hygiene (WASH) practices. Variables included in the creation of the index were: main source of drinking water; type of toilet facilities; water treatment; animal waste; presence of animals in the house; cleanliness of kitchen; protection from flies; washing hands post defecation; washing hands after cleaning the child; washing hands before feeding the child; washing hands before cooking and before eating. This index was further split into three categories (0-3 (poor practices); 4-5 (average practices); 6 or more (good practices)). |
| **Maternal occupation** (baseline) | Three categories were created for maternal occupation: not working; non-agriculture related work; agriculture-related work. |
| **Number of children under 5** (baseline) | This was split into three categories: 1 child; 2 children; 3 or more children. |
| **Number of adult women** (baseline) | Adult women were defined as any women aged 14 or more. The variable was split into three categories: 1; 2; 3 or more. |

**Appendix 3: Variables and indices (continued)**

| **Nutrition-related variables** | |
| --- | --- |
| **Infant and Young Child Minimum Diet Diversity** (endline) | The Infant and Young Child Minimum Dietary Diversity score (IYCMDD) was generated for infants (WHO, 2008), using reported intakes of foods and beverages intakes during the past 24-hours. The calculation was based on 7 pre-determined food groups (i.e. grains, roots and tubers; legumes and nuts; dairy products; flesh foods; eggs; vitamin A rich fruits and vegetables; other fruits and vegetables). Minimum dietary diversity was defined as the consumption of at least 4 groups out of the 7 listed above. |
| **Maternal nutrition knowledge** (endline) | A summative score ranging from 0-11 was created to capture women’s nutrition knowledge. This was based on questions around child feeding practices (i.e. breastfeeding and complementary feeding practices). A knowledge score (range 0-11) was created based on correct (1 point) and incorrect answers (0 point). This was split into 3 categories (i.e. low (0-3 points), middle (4-5 points) and high (≥6 points) nutritional knowledge). |
| **Breastfeeding status** (endline) | This is a binary variable representing current breastfeeding status (yes/no). |
| **Early initiation of breastfeeding** (baseline) | This is a binary variable capturing whether the woman initiated breastfeeding early (yes/no). |
| **Agriculture-related variables** | |
| **Cotton harvesting in the past season** (endline) | Information on cotton harvesting performed in the past season (post-pregnancy) was available. The number of months; number of days and number of hours worked on average on the days worked were collected. Based on this information, a categorical variable was created (no cotton harvesting (i.e. 0 month); 1-2 months; 2 months or more). |
| **Health-related variables** | |
| **Maternal depression** (baseline) | A categorical variable was created for maternal depression (none; mild; moderate or severe depression). |
| **Child diarrhea** (endline) | A binary variable was created to capture child diarrhea experienced in the 2 weeks before the survey. |
| **Child fever** (endline) | A binary variable was created to capture child fever experienced in the 2 weeks before the survey. |
| **Child cough** (endline) | A binary variable was created to capture child cough experienced in the 2 weeks before the survey. |
| **Child vaccination status** (endline) | Binary or categorical variables were created to reflect the vaccination status of infants (BCG (yes/no); polio (no, partially vaccinated, fully vaccinated); Penta (no, partially vaccinated, fully vaccinated); pneumo (no, partially vaccinated, fully vaccinated); measles (yes/no). |

**References**

Coates JS, A; Bilinsky, P. Household Food Insecurity Access Scale (HFIAS) for Measurement of Household Food Access: Indicator Guide (v.3). Washington, D.C.: FHI 360/FANTA. 2007.

World Health Organization (WHO). Indicators for assessing infant and young child feeding practices: conclusions of a consensus meeting held 6-8 November 2007 in Washington DC, USA. 2008.

**Appendix 4: Hypothesized models of pathways related to catch-up growth represented on a directed acyclic graph (DAG)**

*Model 1: testing the relationship between maternal education and catch-up growth*


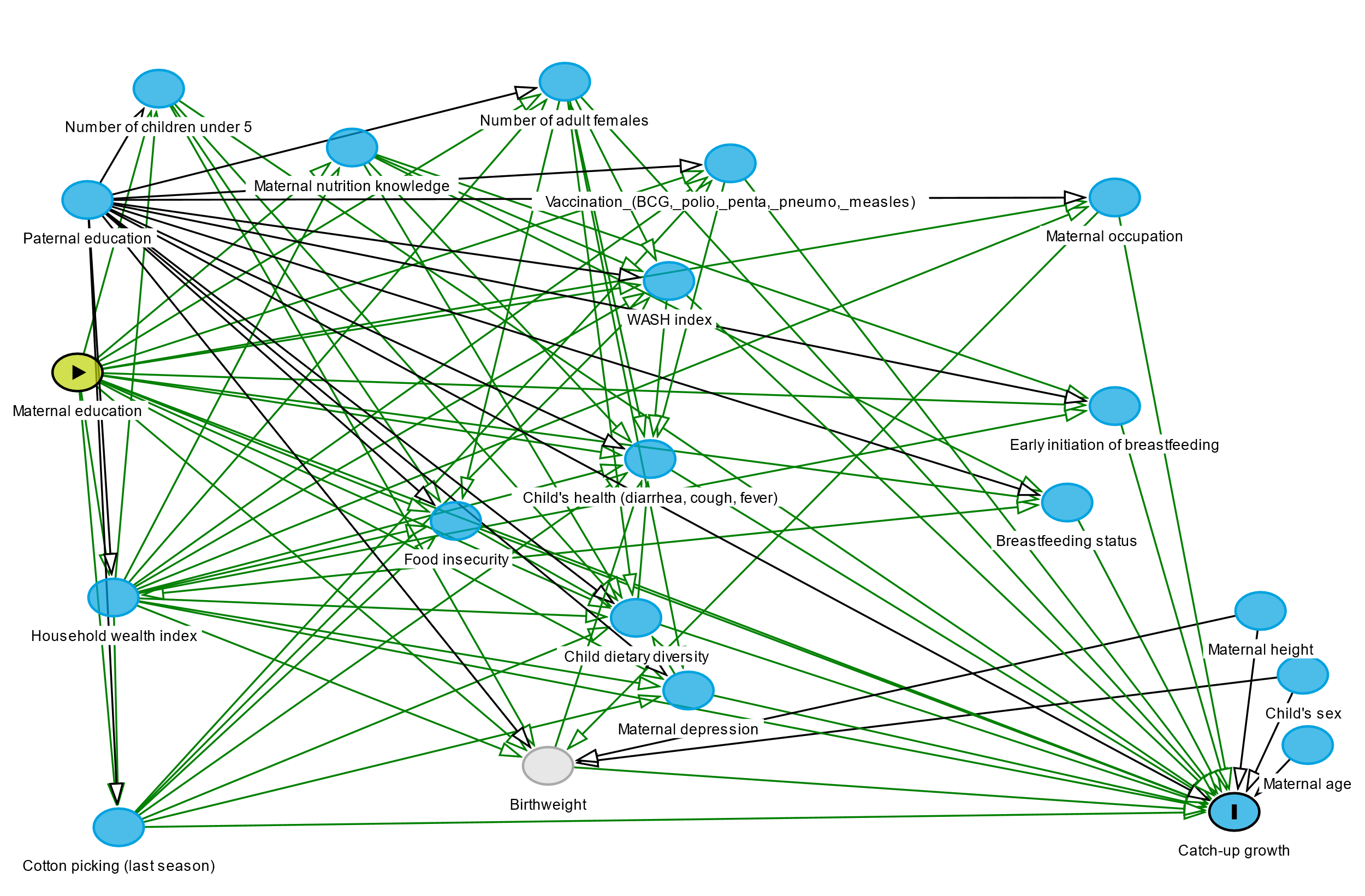


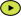
exposure
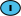
outcome
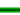
causal path
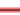
biasing path
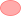
ancestor of exposure and outcome
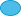
ancestor of outcome
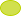
ancestor of exposure

**Appendix 4: Hypothesized models of pathways related to catch-up growth represented on a directed acyclic graph (DAG) (continued)**

*Model 2: testing the relationship between paternal education and catch-up growth*


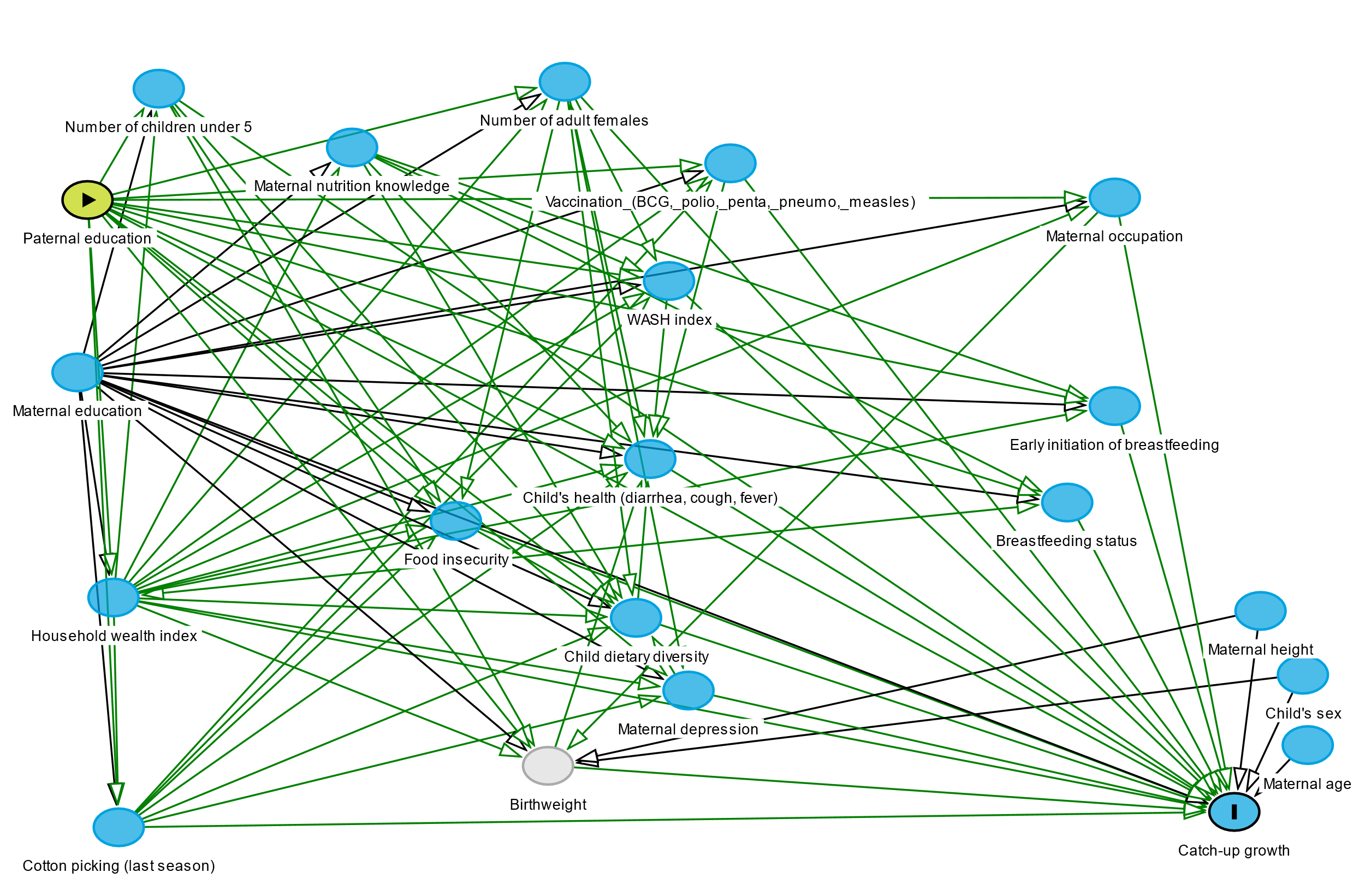


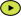
exposure
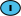
outcome
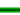
causal path
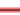
biasing path
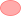
ancestor of exposure and outcome
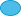
ancestor of outcome
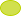
ancestor of exposure

**Appendix 4: Hypothesized models of pathways related to catch-up growth represented on a directed acyclic graph (DAG) (continued)**

*Model 3: testing the relationship between household wealth index and catch-up growth*


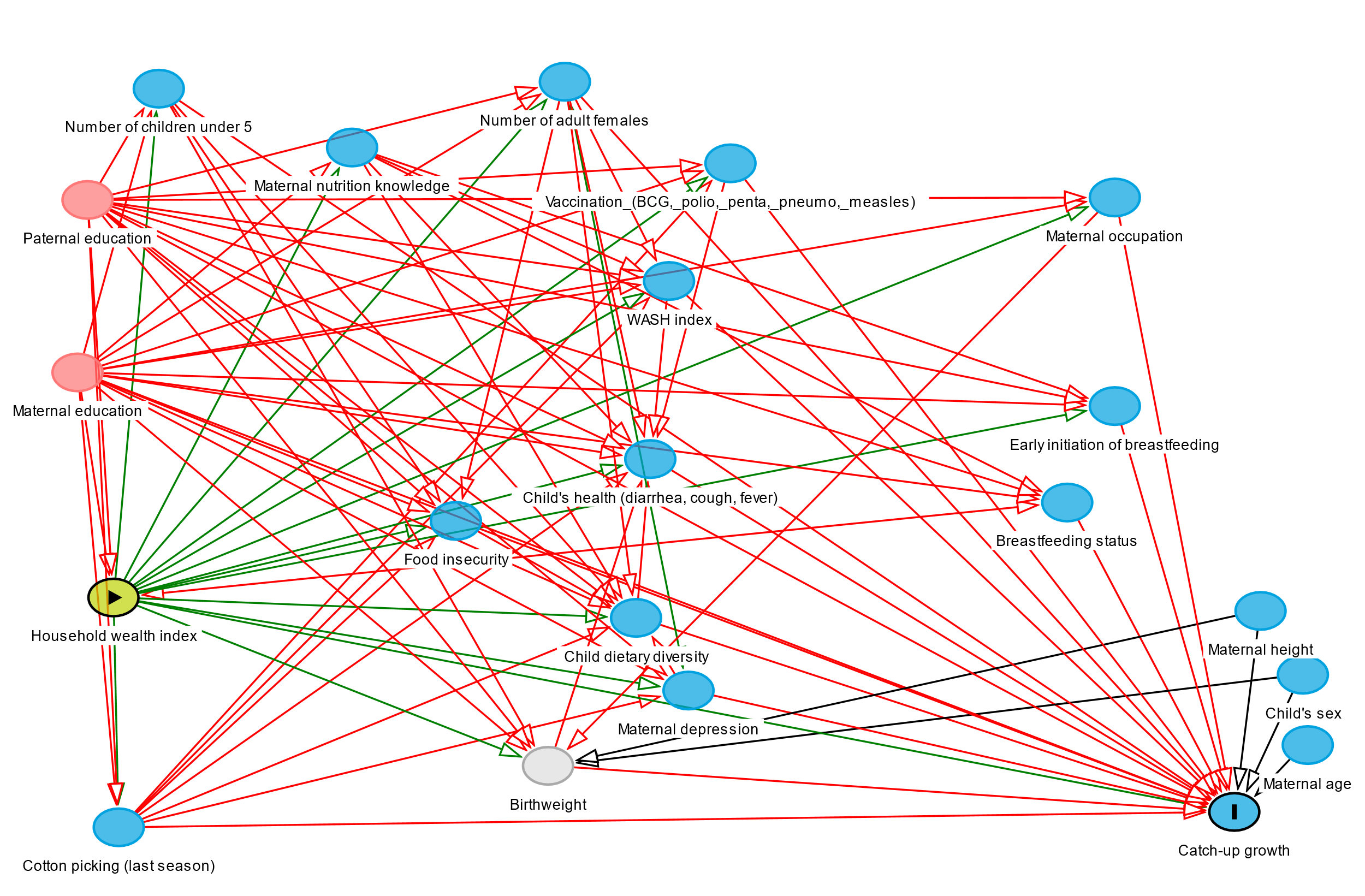


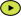
exposure
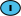
outcome
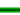
causal path
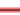
biasing path
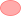
ancestor of exposure and outcome
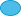
ancestor of outcome
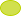
ancestor of exposure

**Appendix 4: Hypothesized models of pathways related to catch-up growth represented on a directed acyclic graph (DAG) (continued)**

*Model 4: testing the relationship between maternal occupation and catch-up growth*


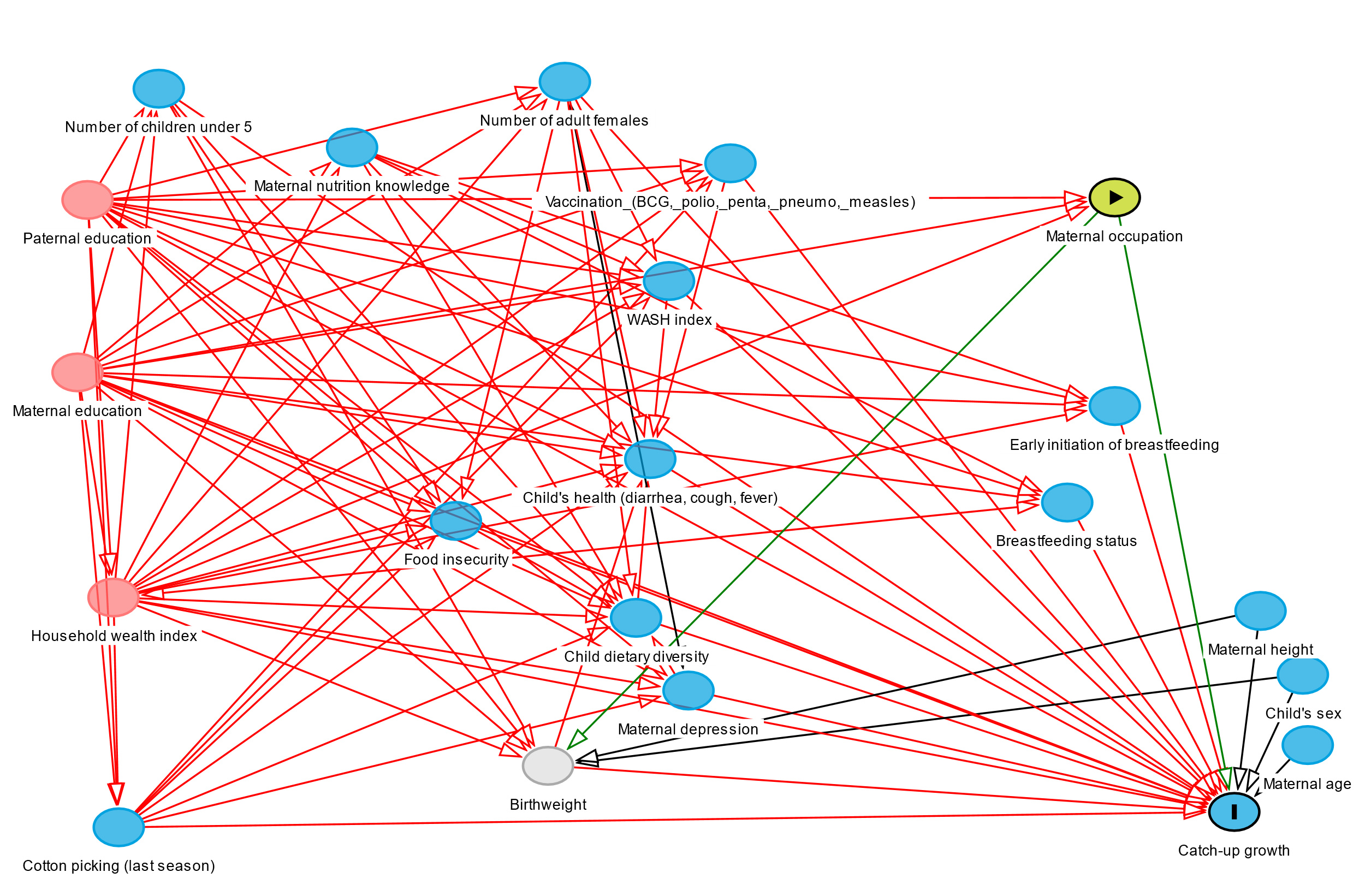


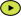
exposure
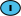
outcome
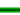
causal path
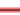
biasing path
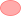
ancestor of exposure and outcome
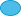
ancestor of outcome
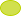
ancestor of exposure

**Appendix 4: Hypothesized models of pathways related to catch-up growth represented on a directed acyclic graph (DAG) (continued)**

*Model 5: testing the relationship between household food insecurity and catch-up growth*


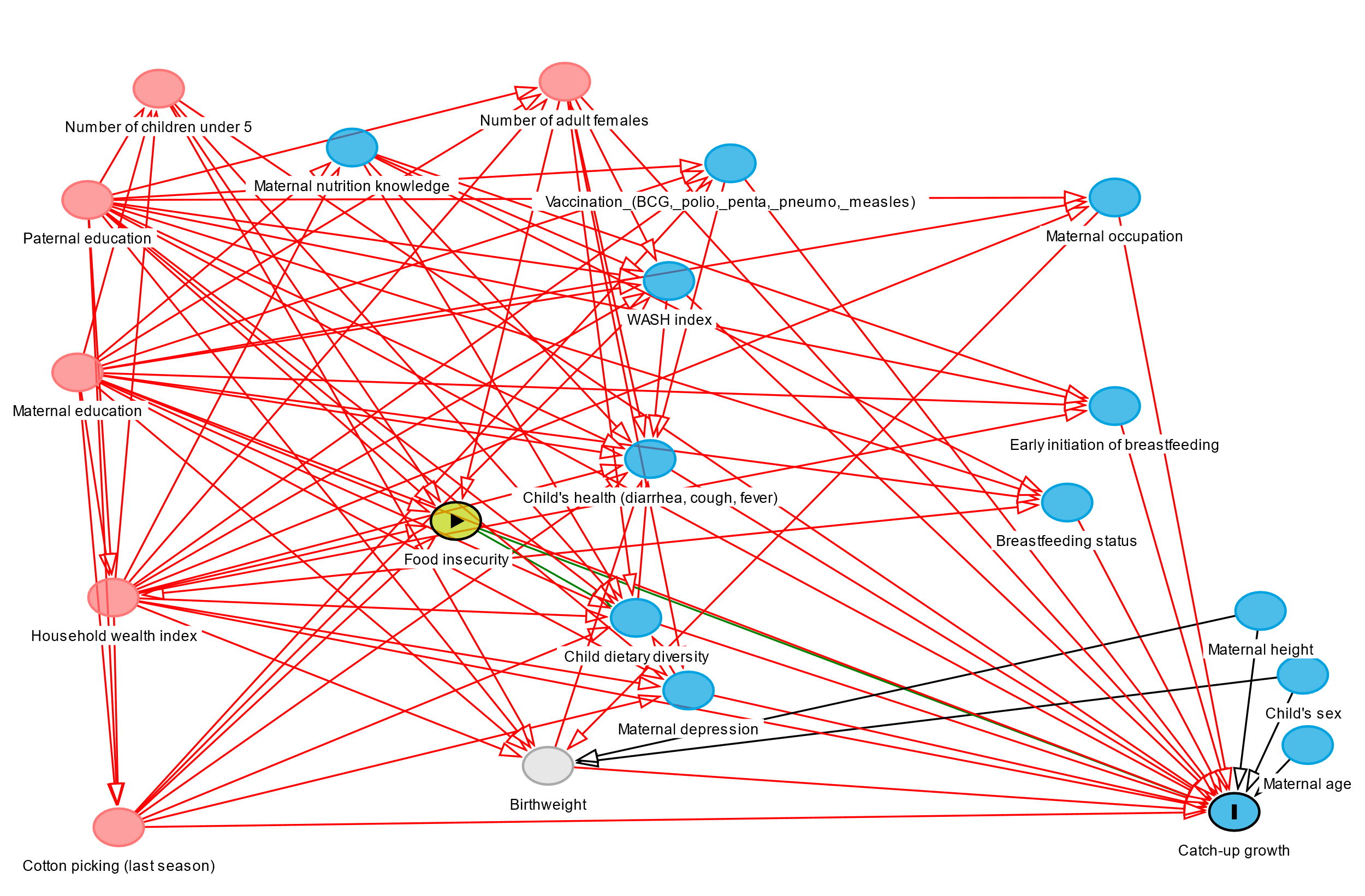


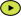
exposure
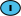
outcome
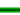
causal path
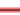
biasing path
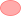
ancestor of exposure and outcome
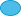
ancestor of outcome
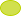
ancestor of exposure

**Appendix 4: Hypothesized models of pathways related to catch-up growth represented on a directed acyclic graph (DAG) (continued)**

*Model 6: testing the relationship between the number of adult females and catch-up growth*


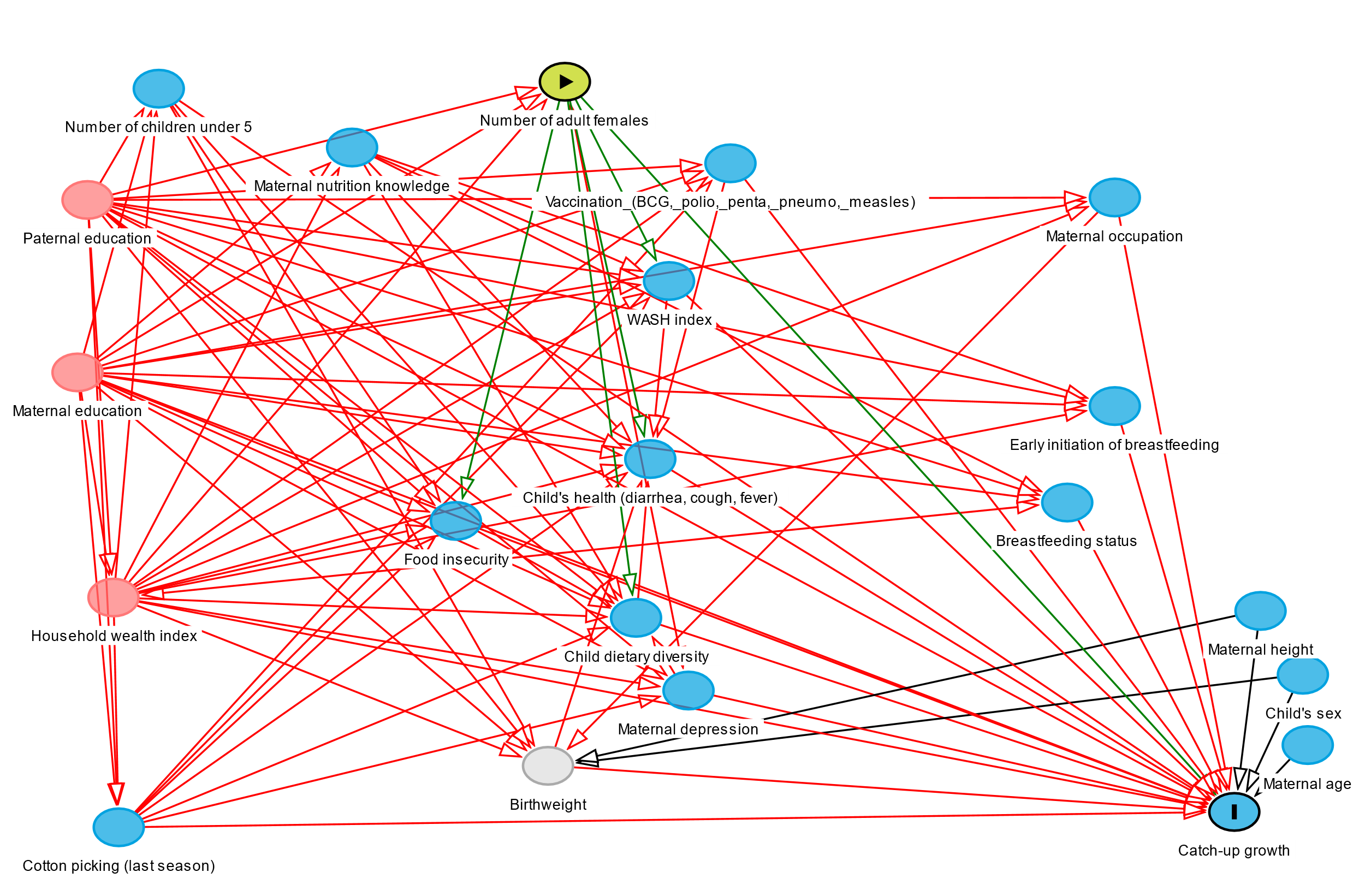


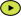
exposure
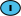
outcome
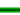
causal path
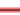
biasing path
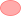
ancestor of exposure and outcome
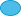
ancestor of outcome
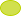
ancestor of exposure

**Appendix 4: Hypothesized models of pathways related to catch-up growth represented on a directed acyclic graph (DAG) (continued)**

*Model 7: testing the relationship between the number of children under 5 and catch-up growth*


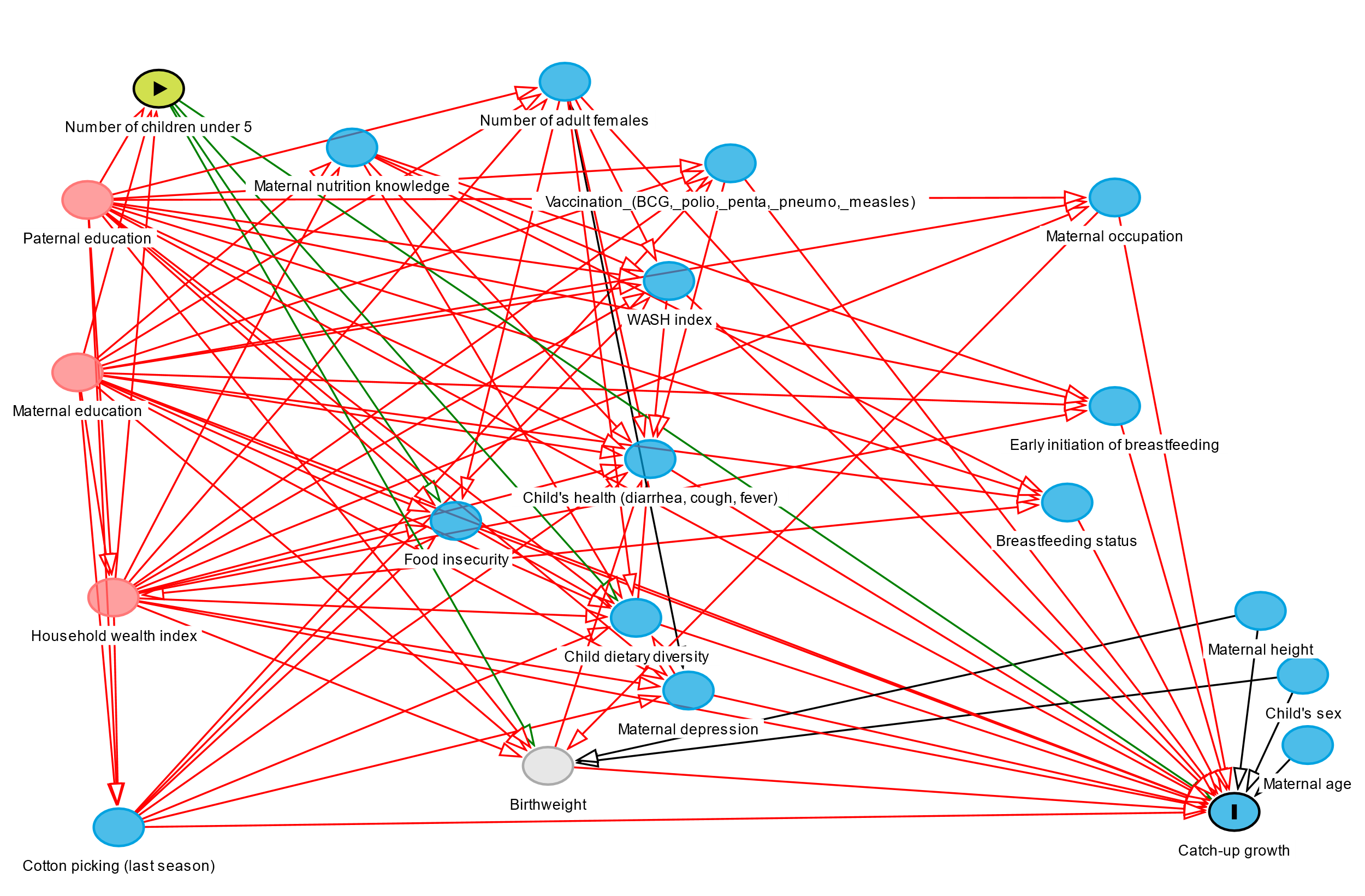


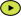
exposure
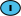
outcome
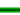
causal path
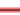
biasing path
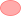
ancestor of exposure and outcome
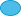
ancestor of outcome
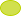
ancestor of exposure

**Appendix 4: Hypothesized models of pathways related to catch-up growth represented on a directed acyclic graph (DAG) (continued)**

*Model 8: testing the relationship between WASH practices and catch-up growth*


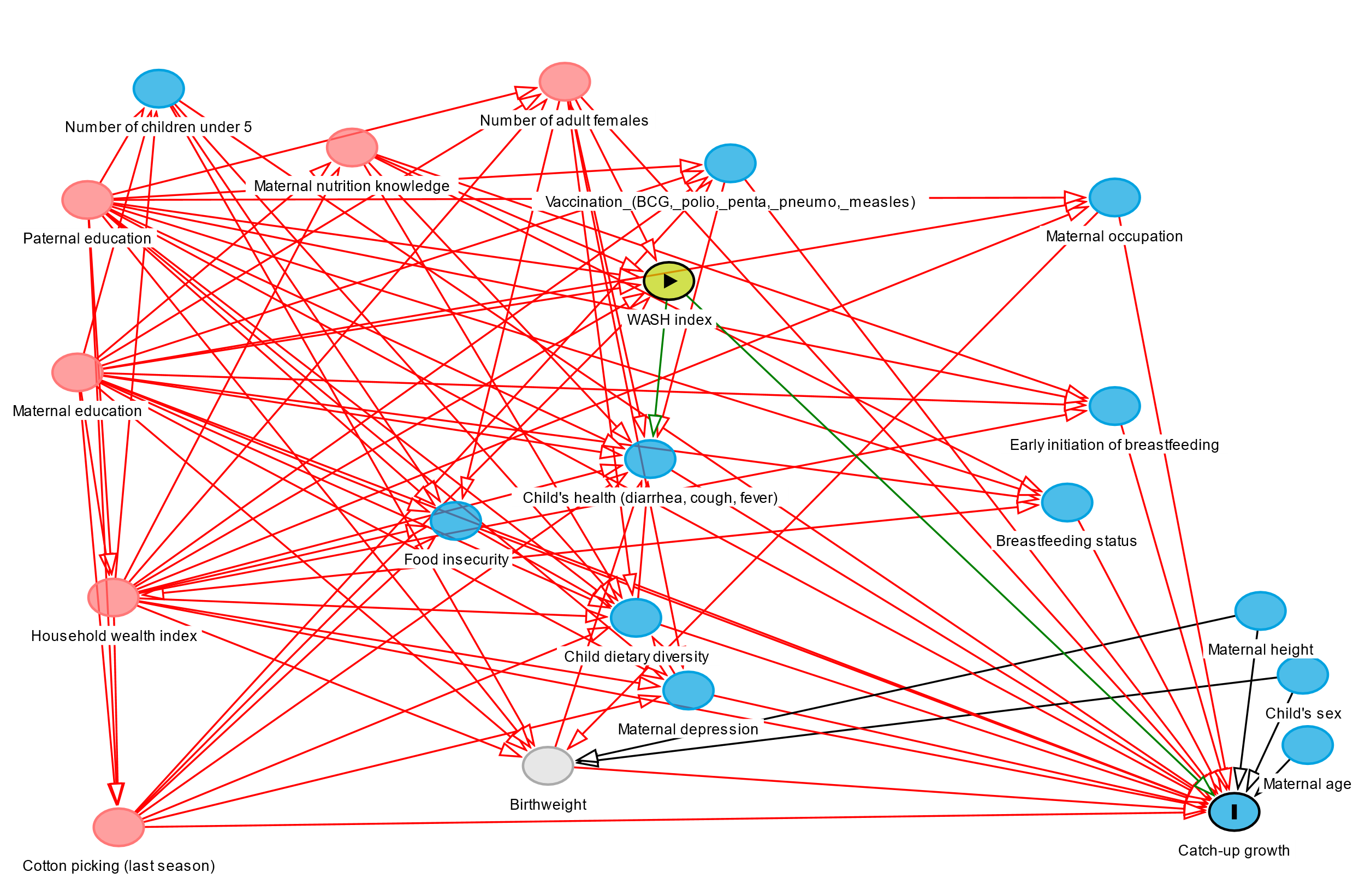


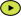
exposure
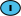
outcome
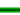
causal path
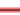
biasing path
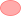
ancestor of exposure and outcome
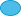
ancestor of outcome
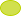
ancestor of exposure

**Appendix 4: Hypothesized models of pathways related to catch-up growth represented on a directed acyclic graph (DAG) (continued)**

*Model 9: testing the relationship between minimum child dietary diversity and catch-up growth*


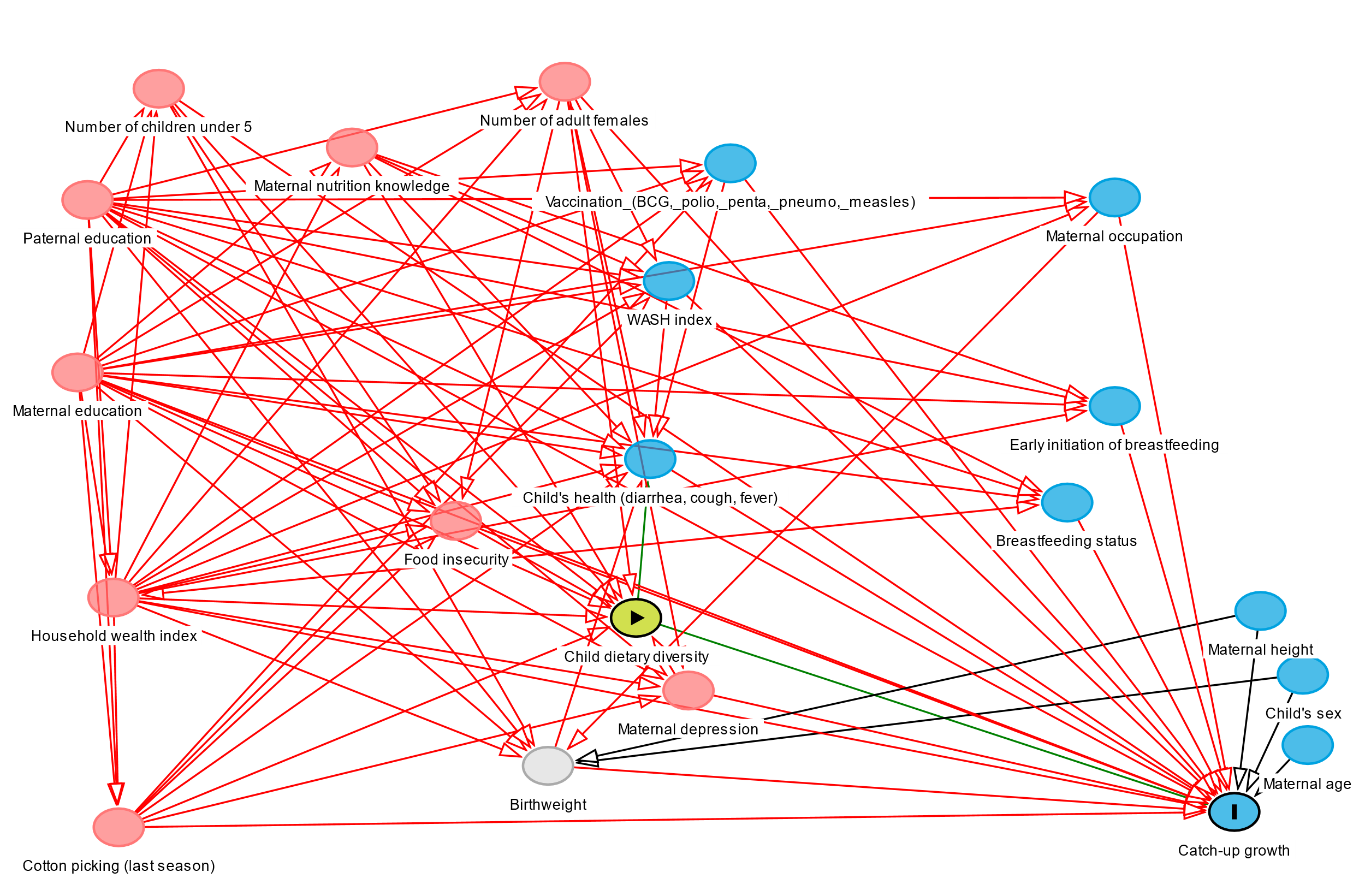


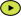
exposure
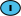
outcome
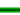
causal path
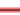
biasing path
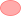
ancestor of exposure and outcome
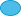
ancestor of outcome
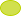
ancestor of exposure

**Appendix 4: Hypothesized models of pathways related to catch-up growth represented on a directed acyclic graph (DAG) (continued)**

*Model 10: testing the relationship between breastfeeding status and catch-up growth*


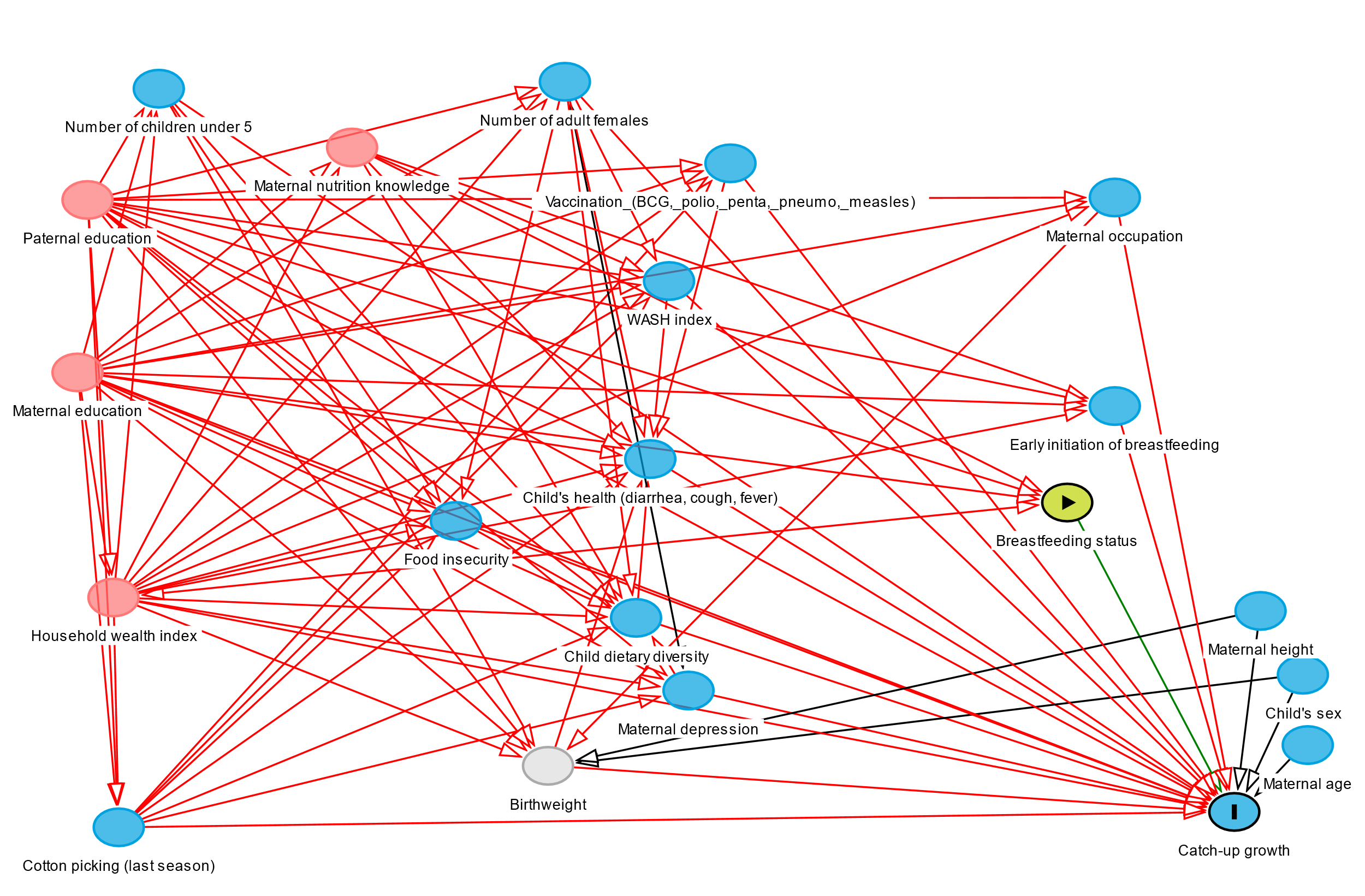


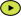
exposure
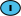
outcome
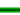
causal path
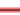
biasing path
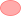
ancestor of exposure and outcome
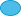
ancestor of outcome
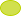
ancestor of exposure

**Appendix 4: Hypothesized models of pathways related to catch-up growth represented on a directed acyclic graph (DAG) (continued)**

*Model 11: testing the relationship between early initiation of breastfeeding and catch-up growth*


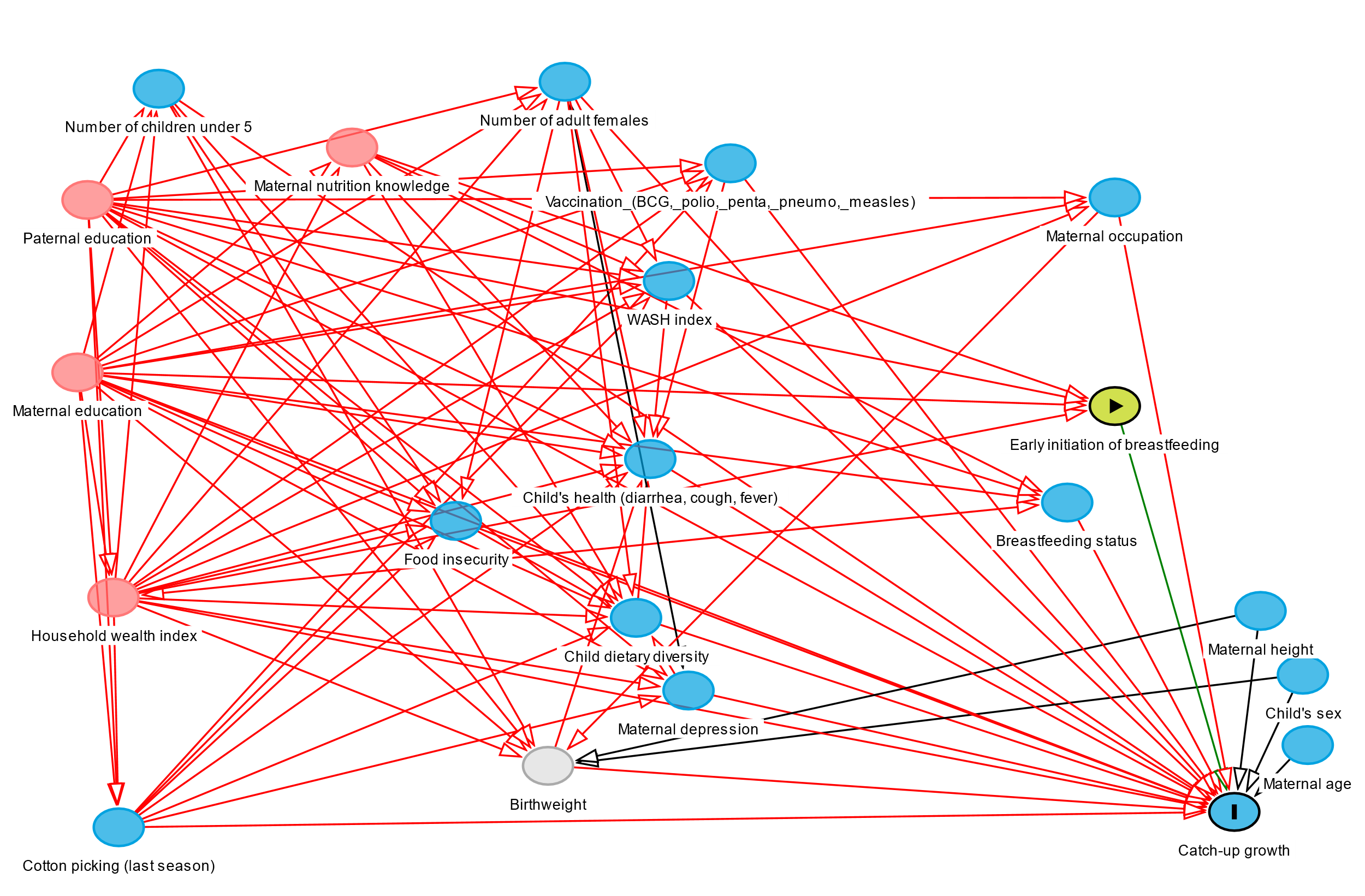


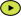
exposure
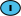
outcome
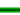
causal path
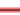
biasing path
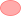
ancestor of exposure and outcome
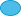
ancestor of outcome
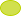
ancestor of exposure

**Appendix 4: Hypothesized models of pathways related to catch-up growth represented on a directed acyclic graph (DAG) (continued)**

*Model 12: testing the relationship between maternal nutrition knowledge and catch-up growth*


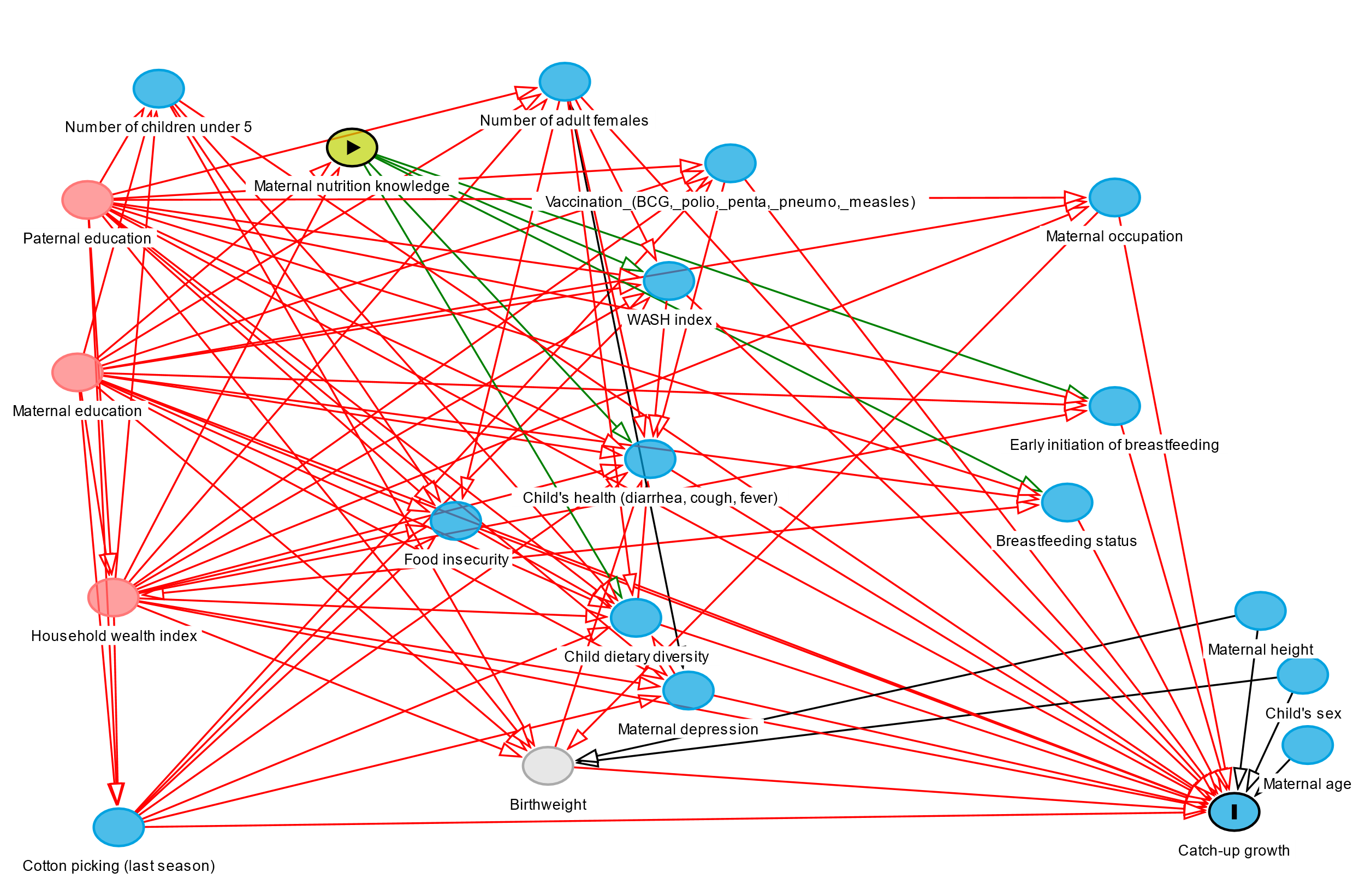


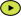
exposure
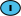
outcome
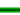
causal path
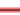
biasing path
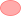
ancestor of exposure and outcome
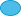
ancestor of outcome
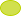
ancestor of exposure

**Appendix 4: Hypothesized models of pathways related to catch-up growth represented on a directed acyclic graph (DAG) (continued)**

*Model 13: testing the relationship between cotton harvesting (post pregnancy) and catch-up growth*


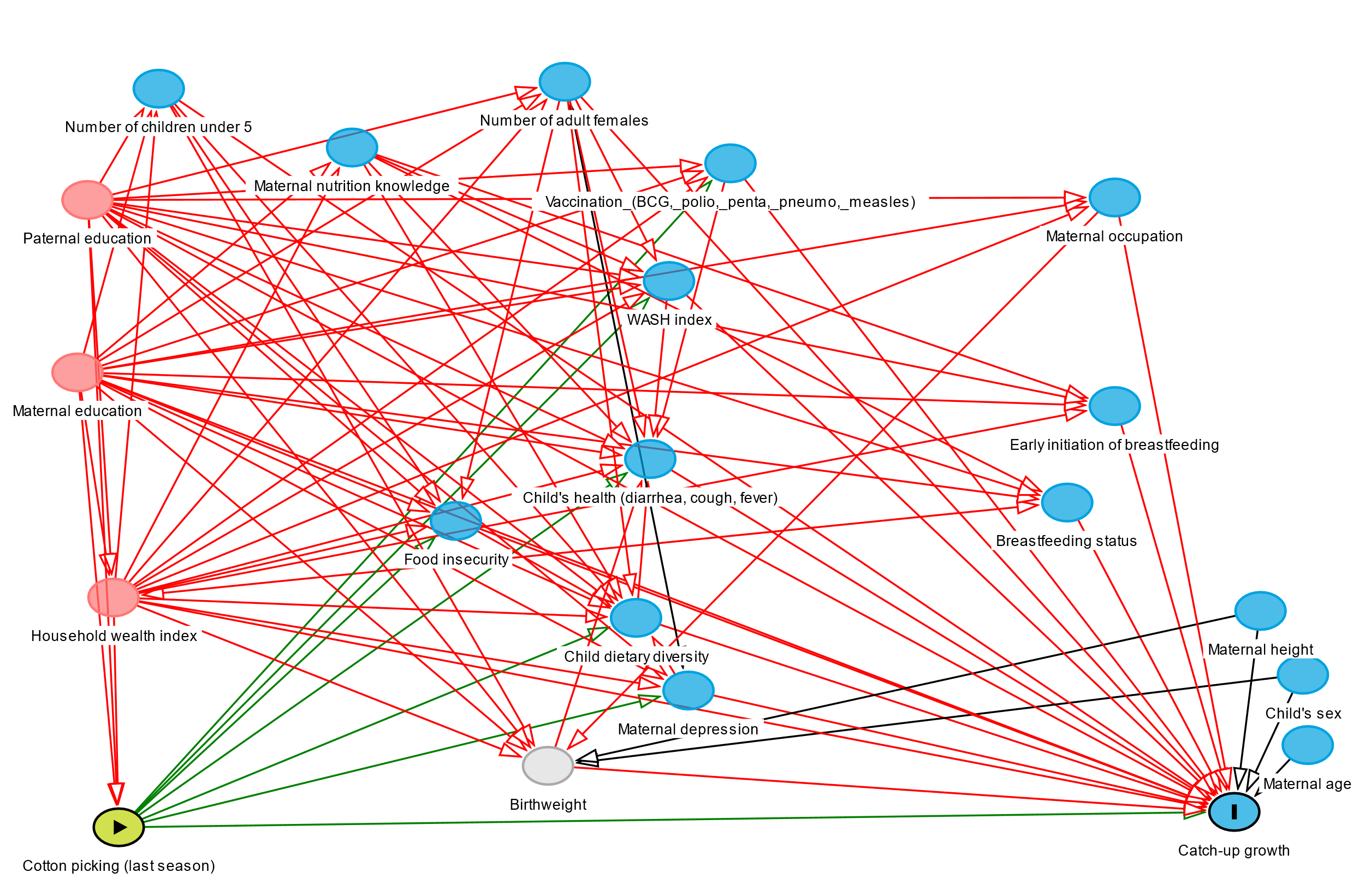


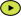
exposure
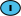
outcome
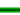
causal path biasing path ancestor of exposure and outcome ancestor of outcome ancestor of exposure

**Appendix 5: Sample characteristics for infants with and without endline outcome data**

|  | **Those with complete outcome data (n=1008)** | **Those with missing outcome data**  **(n=155)** | **Difference***  **(complete–missing)** |
| --- | --- | --- | --- |
| ***Infant characteristics*** | | | |
| Sex (female)  (% (n)) | 50.1% (505) | 50.0%  (14/28) | 0.1%; p=0.99 |
| Length at baseline (cm) (mean, SD) | 52.7 (3.6) | 50.8 (4.3)  (n=137) | 1.9 (1.2;2.5) |
| Length-for-age z score at baseline  (mean, SD) | -1.8 (1.4) | -2.3 (1.5)  (n=128) | 0.5 (0.2;0.71) |
| Stunted at baseline  (% (n)) | 44.3% (446) | 53.9%  (69/128) | -9.7%; p=0.04 |
| ***Maternal characteristics*** | | | |
| Age at visit 1  (years) (mean; SD) | 28.0 (6.4)  (n=170) | 27.0 (6.6)  (n=140) | 1.0 (-0.2; 2.1) |
| BMI at visit 1 (kg/m^2^) (median; IQR) | 20.6  (18.8;22.6) | 20.1 (18.1;22.3) (n=140) | -0.5 (-1.1;0.2) |
| Multiparous  (% (n)) | 78.4% (790) | 77.6%  (114/147) | 0.8%; p=0.8 |
| Educated (yes) (% (n)) | 20.0% (202) | 12.9%  (19/147) | 7.1%; p=0.04 |
| Top tertile nutritional knowledge score (% (n)) | 28.9% (290/1002) | 15.2% (5/33) | 13.8%; p=0.1 |
| ***Household characteristics*** | | | |
| Household food insecurity score (median (IQR)) | 7 (0;13)  (n=985) | 8 (2;12)  (n=33) | 1 (-3.5;5.5) |
| Minimum dietary diversity score (yes) (% (n)) | 9.3% (93/1002) | 6.1% (2/33) | 3.2%; p=0.5 |
| Top tertile socioeconomic status (% (n)) | 33.5% (328/979) | 31.7% (45/142) | 1.8%; p=0.7 |
| ≥3 adult females  (n (%)) | 68.5% (690) | 61.9% (96/155) | 6.5%; p=0.1 |
| ≥3 children less than 5 years old  (n (%)) | 33.4% (337) | 36.8% (57/155) | -3.3%; p=0.4 |

_*categorical variables: chi_^2^ _test; continuous variables: studentised t-tests for mean differences (95% CI) and quantile regression (50th centile) for differences in medians_

**Appendix 6: Sensitivity analyses**

*Replacing catch-up=. with catch-up=0*

| **Univariable analysis** | | | | |
| --- | --- | --- | --- | --- |
|  | **OR** | **95% CI** | **p** | **overall p value** |
| **Maternal age** | 1 | 0.97-1.02 | p=0.079 |  |
| **Maternal height** | 1.09 | 1.05-1.12 | p<0.001 |  |
| Sex | 0.83 | 0.61-1.13 | p=0.235 |  |
| **Maternal education** |  |  |  |  |
| *primary* | 2.45 | 1.59-3.76 | p<0.001 | p<0.001 |
| *middle* | 2.67 | 1.43-4.98 | p=0.002 |  |
| **Paternal education** |  |  |  |  |
| *primary* | 1.06 | 0.69-1.64 | p=0.777 | p=0.01 |
| *middle* | 1.56 | 1.14-2.13 | p=0.006 |  |
| **Household wealth index** |  |  |  |  |
| *poor* | 1.59 | 0.92-2.76 | p=0.1 | p<0.001 |
| *middle* | 2.13 | 1.14-3.98 | p=0.018 |  |
| *wealthy* | 2.25 | 1.29-3.93 | p=0.004 |  |
| *wealthiest* | 4.13 | 2.39-7.15 | p<0.001 |  |
| **Maternal occupation** |  |  |  |  |
| *Non-agricultural related* | 1 | 0.66-1.51 | p=0.992 | p=0.005 |
| *Agricultural related* | 0.62 | 0.45-0.85 | p=0.003 |  |
| **Household Food Insecurity** |  |  |  |  |
| *mildly* | 0.7 | 0.40-1.24 | p=0.22 | p=0.002 |
| *moderately* | 0.65 | 0.44-0.96 | p=0.03 |  |
| *severely* | 0.49 | 0.34-0.71 | p<0.001 |  |
| **Number of adult females** |  |  |  |  |
| *2* | 2.11 | 1.44-3.11 | p<0.001 | p=0.001 |
| *≥3* | 1.61 | 1.10-2.34 | p=0.014 |  |
| **Number of children under 5** |  |  |  |  |
| *2* | 0.62 | 0.45-0.87 | p=0.005 | p=0.0001 |
| *≥3* | 0.43 | 0.30-0.64 | p<0.001 |  |
| **WASH index** |  |  |  |  |
| *4-5 points* | 1.47 | 1.08-2.12 | p=0.015 | p=0.001 |
| *≥6 points* | 2.07 | 1.39-3.09 | p<0.001 |  |
| **Minimum dietary diversity** |  |  |  |  |
| *yes* | 1.83 | 1.17-2.86 | p=0.009 |  |
| **Breastfeeding** |  |  |  |  |
| *yes* | 0.59 | 0.43-0.83 | p=0.002 |  |
| **Early initiation of breastfeeding** |  |  |  |  |
| *yes* | 1.01 | 0.75-1.35 | p=0.96 |  |
| **Nutrition knowledge score** |  |  |  |  |
| *4-5 points* | 1.26 | 0.94-1.70 | p=0.127 | p=0.125 |
| ≥6 points | 1.53 | 1.01-2.31 | p=0.043 |  |
| **Cotton picking in the past season** |  |  |  |  |
| *1-2 months* | 1.05 | 0.67-1.64 | p=0.84 | p=0.025 |
| *≥2 months* | 0.6 | 0.40-0.91 | p=0.015 |  |

**Appendix 6: Sensitivity analyses (continued)**

*Replacing catch-up=. with catch-up=0*

| **Multivariable analysis** | | | | |
| --- | --- | --- | --- | --- |
|  | **OR** | **95% CI** | **p** | **overall p value** |
| **Maternal education** |  |  |  |  |
| *Primary* | 2.52 | 1.65-3.85 | p<0.001 | p<0.001 |
| *Middle* | 2.49 | 1.33-4.67 | p=0.004 |  |
| **Paternal education** |  |  |  |  |
| *Primary* | 1.08 | 0.69-1.70 | p=0.72 | p=0.0374 |
| *Middle* | 1.49 | 1.07-2.06 | p=0.017 |  |
| **Household wealth index** |  |  |  |  |
| *Poor* | 1.78 | 1.02-3.11 | p=0.041 | p=0.001 |
| *Middle* | 1.96 | 0.98-3.92 | p=0.057 |  |
| *Wealthy* | 2.18 | 1.18-4.00 | p=0.012 |  |
| *Wealthiest* | 3.69 | 1.94-6.99 | p<0.001 |  |
| **Maternal occupation** |  |  |  |  |
| *Non-agricultural related* | 1.07 | 0.66-1.75 | p=0.775 | p=0.522 |
| *Agricultural related* | 0.85 | 0.60-1.19 | p=0.34 |  |
| **Household Food Insecurity** |  |  |  |  |
| *Mildly* | 0.65 | 0.35-1.22 | p=0.179 | p=0.371 |
| *Moderately* | 0.83 | 0.52-1.32 | p=0.443 |  |
| *Severely* | 0.73 | 0.48-1.12 | p=0.147 |  |
| **Number of adult females** |  |  |  |  |
| *2* | 1.93 | 1.27-2.92 | p=0.002 | p=0.007 |
| *≥3* | 1.28 | 0.86-1.91 | p=0.215 |  |
| **Number of children under 5** |  |  |  |  |
| *2* | 0.65 | 0.46-0.92 | p=0.014 | p=0.001 |
| *≥3* | 0.45 | 0.30-0.68 | p<0.001 |  |
| **WASH index** |  |  |  |  |
| *4-5 points* | 1.22 | 0.85-1.75 | p=0.277 | p=0.196 |
| *≥6 points* | 1.41 | 0.96-2.06 | p=0.078 |  |
| **Minimum dietary diversity** |  |  |  |  |
| *yes* | 1.42 | 0.77-2.64 | p=0.265 |  |
| **Breastfeeding** |  |  |  |  |
| *yes* | 0.6 | 0.41-0.89 | p=0.01 |  |
| **Early initiation of breastfeeding** |  |  |  |  |
| *yes* | 1.15 | 0.86-1.56 | p=0.348 |  |
| **Nutrition knowledge score** |  |  |  |  |
| *4-5 points* | 1.19 | 0.84-1.67 | p=0.323 | p=0.5592 |
| ≥6 points | 1.08 | 0.68-1.70 | p=0.757 |  |
| **Cotton picking in the past season** |  |  |  |  |
| *1-2 months* | 1.44 | 0.93-2.21 | p=0.1 | p=0.031 |
| *≥2 months* | 0.75 | 0.49-1.14 | p=0.178 |  |

**Appendix 6: Sensitivity analyses (continued)**

*Replacing catch-up=. with catch-up=1*

| **Univariable analysis** | | | | |
| --- | --- | --- | --- | --- |
|  | **OR** | **95% CI** | **p** | **overall p value** |
| **Maternal age** | 1 | 0.98-1.02 | p=0.87 |  |
| **Maternal height** | 1.06 | 1.03-1.10 | p<0.001 |  |
| Sex | 0.83 | 0.61-1.12 | p=0.23 |  |
| **Maternal education** |  |  |  |  |
| *primary* | 2.27 | 1.54-3.33 | p<0.001 | p<0.001 |
| *middle* | 2.29 | 1.25-4.18 | p=0.007 |  |
| **Paternal education** |  |  |  |  |
| *primary* | 1.01 | 0.67-1.52 | p=0.955 | p=0.035 |
| *middle* | 1.38 | 1.03-1.86 | p=0.033 |  |
| **Household wealth index** |  |  |  |  |
| *poor* | 1.33 | 0.82-2.15 | p=0.249 | p<0.001 |
| *middle* | 1.6 | 0.92-2.75 | p=0.095 |  |
| *wealthy* | 1.81 | 1.10-2.97 | p=0.019 |  |
| *wealthiest* | 3.02 | 1.88-4.86 | p<0.001 |  |
| **Maternal occupation** |  |  |  |  |
| *Non-agricultural related* | 0.99 | 0.66-1.47 | p=0.944 | p=0.043 |
| *Agricultural related* | 0.71 | 0.53-0.94 | p=0.019 |  |
| **Household Food Insecurity** |  |  |  |  |
| *mildly* | 0.78 | 0.44-1.35 | p=0.371 | p=0.002 |
| *moderately* | 0.68 | 0.47-0.98 | p=0.041 |  |
| *severely* | 0.54 | 0.40-0.75 | p<0.001 |  |
| **Number of adult females** |  |  |  |  |
| *2* | 1.79 | 1.27-2.51 | p=0.001 | p=0.002 |
| *≥3* | 1.46 | 1.07-2.00 | p=0.017 |  |
| **Number of children under 5** |  |  |  |  |
| *2* | 0.65 | 0.47-0.89 | p=0.008 | p=0.0001 |
| *≥3* | 0.46 | 0.32-0.66 | p<0.001 |  |
| **WASH index** |  |  |  |  |
| *4-5 points* | 1.44 | 1.07-1.94 | p=0.016 | p=0.004 |
| *≥6 points* | 1.83 | 1.26-2.66 | p=0.002 |  |
| **Minimum dietary diversity** |  |  |  |  |
| *yes* | 1.66 | 1.07-2.57 | p=0.023 |  |
| **Breastfeeding** |  |  |  |  |
| *yes* | 0.61 | 0.44-0.85 | p=0.003 |  |
| **Early initiation of breastfeeding** |  |  |  |  |
| *yes* | 0.93 | 0.70-1.22 | p=0.590 |  |
| **Nutrition knowledge score** |  |  |  |  |
| *4-5 points* | 1.33 | 0.95-1.86 | p=0.095 | p=0.20 |
| ≥6 points | 1.37 | 0.93-2.03 | p=0.11 |  |
| **Cotton picking in the past season** |  |  |  |  |
| *1-2 months* | 1.05 | 0.71-1.55 | p=0.807 | p=0.0496 |
| *≥2 months* | 0.64 | 0.43-0.95 | p=0.025 |  |

**Appendix 6: Sensitivity analyses (continued)**

*Replacing catch-up=. with catch-up=1 (continued)*

| **Multivariable analysis** | | | | |
| --- | --- | --- | --- | --- |
|  | **OR** | **95% CI** | **p** | **overall p value** |
| **Maternal education** |  |  |  |  |
| *primary* | 2.28 | 1.55-3.46 | p<0.001 | p<0.001 |
| *middle* | 2.18 | 1.18-4.01 | p=0.012 |  |
| **Paternal education** |  |  |  |  |
| *primary* | 1.06 | 0.69-1.62 | p=0.8 | p=0.139 |
| *middle* | 1.35 | 0.98-1.86 | p=0.068 |  |
| **Household wealth index** |  |  |  |  |
| *poor* | 1.48 | 0.88-2.48 | p=0.139 | p=0.01 |
| *middle* | 1.47 | 0.79-2.75 | p=0.227 |  |
| *wealthy* | 1.69 | 0.96-2.97 | p=0.067 |  |
| *wealthiest* | 2.75 | 1.51-5.01 | p=0.001 |  |
| **Maternal occupation** |  |  |  |  |
| *Non-agricultural related* | 1.08 | 0.67-1.75 | p=0.743 | p=0.746 |
| *Agricultural related* | 0.92 | 0.66-1.27 | p=0.594 |  |
| **Household Food Insecurity** |  |  |  |  |
| *mildly* | 0.77 | 0.42-1.41 | p=0.4 | p=0.713 |
| *moderately* | 0.86 | 0.56-1.34 | p=0.519 |  |
| *severely* | 0.83 | 0.58-1.20 | p=0.32 |  |
| **Number of adult females** |  |  |  |  |
| *2* | 1.67 | 1.14-2.43 | p=0.008 | p=0.026 |
| *≥3* | 1.19 | 0.84-1.69 | p=0.319 |  |
| **Number of children under 5** |  |  |  |  |
| *2* | 0.64 | 0.46-0.89 | p=0.008 | p<0.001 |
| *≥3* | 0.43 | 0.29-0.63 | p<0.001 |  |
| **WASH index** |  |  |  |  |
| *4-5 points* | 1.26 | 0.88-1.79 | p=0.203 | p=0.239 |
| *≥6 points* | 1.35 | 0.93-1.96 | p=0.109 |  |
| **Minimum dietary diversity** |  |  |  |  |
| *yes* | 1.31 | 0.72-2.40 | p=0.379 |  |
| **Breastfeeding** |  |  |  |  |
| *yes* | 0.6 | 0.41-0.87 | p=0.007 |  |
| **Early initiation of breastfeeding** |  |  |  |  |
| *yes* | 1.08 | 0.81-1.44 | p=0.59 |  |
| **Nutrition knowledge score** |  |  |  |  |
| *4-5 points* | 1.21 | 0.86-1.70 | p=0.265 | p=0.352 |
| ≥6 points | 0.98 | 0.63-1.53 | p=0.945 |  |
| **Cotton picking in the past season** |  |  |  |  |
| *1-2 months* | 1.31 | 0.89-1.91 | p=0.171 | p=0.1214 |
| *≥2 months* | 0.8 | 0.52-1.21 | p=0.282 |  |

**Appendix 7: Sample flow chart**
